# N‐Acetyl‐l‐cysteine restores reproductive defects caused by *Ggt1* deletion in mice

**DOI:** 10.1002/ctm2.510

**Published:** 2021-08-25

**Authors:** Ling Wang, Jinhua Tang, Jiawei Zhou, Lihua Zhu, Feng Tan, Yaru Chen, Lei Wang, Huibin Song, Yiliang Miao, Shuqi Mei, Fenge Li

**Affiliations:** ^1^ Key Lab of Swine Genetics and Breeding of Ministry of Agriculture and Rural Affairs & Key Laboratory of Agricultural Animal Genetics Breeding and Reproduction of Ministry of Education Huazhong Agricultural University Wuhan P. R. China; ^2^ Hubei Academy of Agricultural Sciences Institute of Animal Science and Veterinary Medicine Wuhan P. R. China; ^3^ The Cooperative Innovation Center for Sustainable Pig Production Huazhong Agricultural University Wuhan P. R. China

Dear Editor,

Polycystic ovary syndrome (PCOS) is an autoimmune disease that is characterized by follicular growth arrest and chronic anovulation and linked with female infertility in 5%–20% of reproductive‐aged women.^1^ γ‐Glutamyltranspeptidase (GGT1), which is one of the membrane‐associated enzymes in mammalian cells, may lead to an altered glucose metabolism and abnormal lipid profile that are main clinical phenotypes of PCOS.^2^ Here, we discovered that *Ggt1‐*deficient female mice had similar phenotypes to those of women with PCOS. The reproductive defects in *Ggt1*‐null mice were restored by N‐acetyl‐l‐cysteine (NAC), which is an antioxidant commonly used in the treatment of PCOS patients.^3^

*Ggt1^−/−^* mice generated by CRISPR/Cas9 system (Figure 1A,B) showed smaller body size and ovaries (Figure 1C,D). *Ggt1^−/−^* females were anovulatory and infertile with elevated testosterone and LH/FSH levels and decreased prostaglandin (PGE2) levels, which are similar to PCOS in women (Figure 1E–M). Moreover, *Ggt1^−/−^* mice showed abnormal follicular development with no antral follicles and more atretic follicles, and insensitivity to exogenous gonadotropins stimulation (Figure 1N–W). Granulosa cells (GCs) provide the microenvironment required for developing oocytes and follicles, and ovarian granulosa cell apoptosis is the main cause of follicular atresia.^4^ There were no significant changes in apoptosis and cell proliferation between 3‐week‐old *Ggt1^+/+^* and *Ggt1^−/−^* ovaries (Figure S1A,B). But GCs with more apoptosis and less proliferation were found in 8‐week‐old *Ggt1^−/−^* ovaries compared with the *Ggt1^+/+^* mice (Figure 2A–E).

**FIGURE 1:**
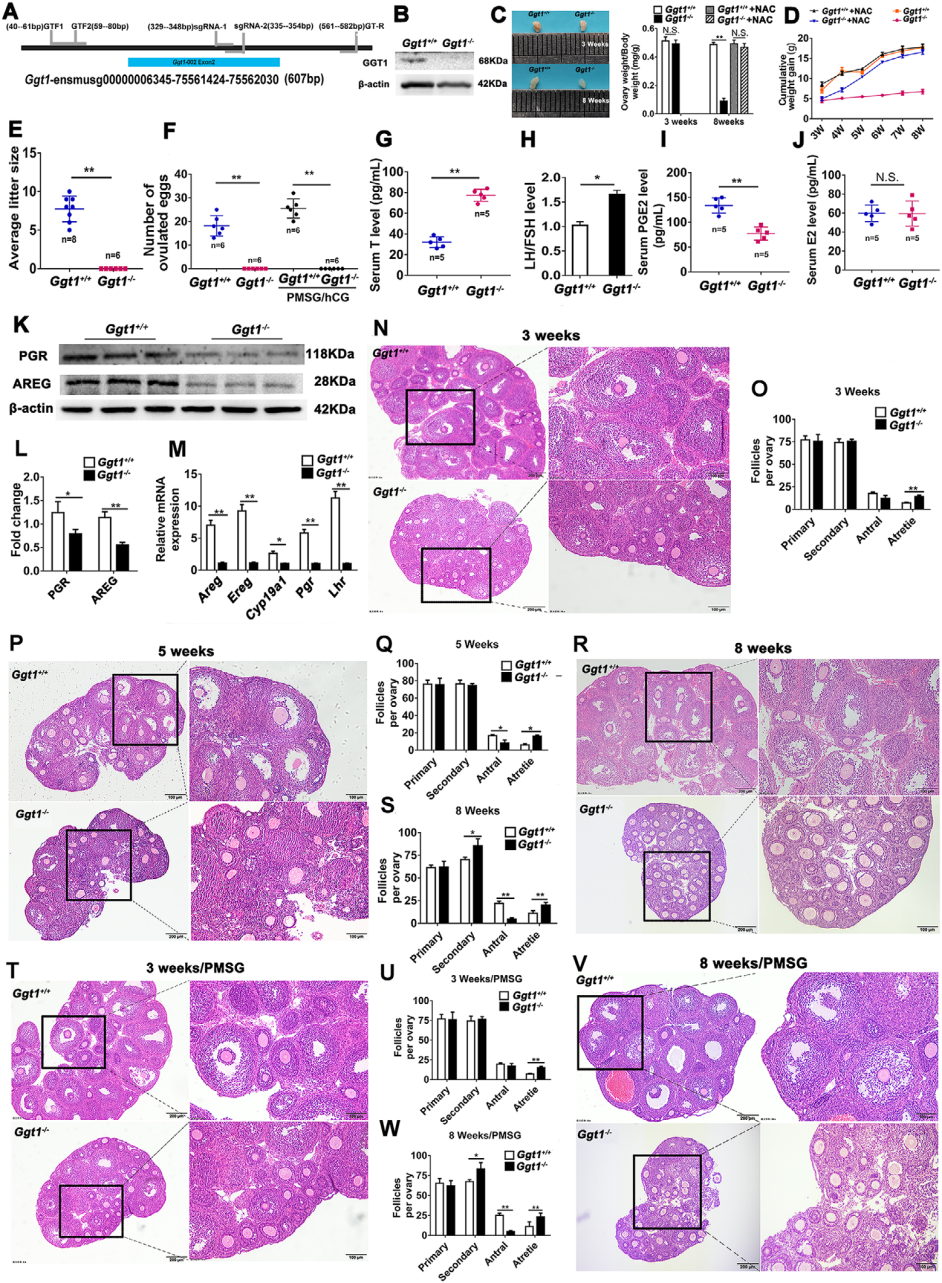
*Ggt1*‐null mice have analogous phenotypes to the clinical symptoms of PCOS patients. (A) The schematic diagram of constructing a *Ggt1* knockout mouse. (B) Western blotting analysis of GGT1 protein level in ovaries from *Ggt1^+/+^* and *Ggt1^−/−^* mice. (C) Representative images of ovaries and the ratio of the ovary to body weight from 3‐ and 8‐week‐old *Ggt1^+/+^* and *Ggt1^−/−^* mice. (D) The cumulative weight gain of *Ggt1^+/+^*, *Ggt1^−/−^*, *Ggt1^+/+^* +NAC and *Ggt1^−/−^* +NAC mice. (E) The average litter size and (F) the number of ovulated eggs were recorded in *Ggt1^+/+^* and *Ggt1^−/−^* females. (G) Testosterone, (H) luteinizing hormone/follicle‐stimulating hormone, (I) prostaglandin, and (J) estrogen levels were observed in *Ggt1^+/+^* and *Ggt1^−/−^* mice. (G–J) Sera collected from 8‐week‐old *Ggt1^+/+^* and *Ggt1^−/−^* mice were examined for hormone level. Testosterone, luteinizing hormone/follicle‐stimulating hormone, prostaglandin, and estrogen are abbreviated as T, LH/FSH, PGE2, and E2, respectively. (K and L) PGR and AREG protein levels in 8‐week‐old *Ggt1^+/+^* and *Ggt1^−/−^* ovaries. (M) *Areg*, *Ereg*, *Cyp19a1*, *Pgr*, and *Lhr* mRNA levels in 8‐week‐old *Ggt1^+/+^* and *Ggt1^−/−^* ovaries. Histological sections were observed in the ovaries from *Ggt1^+/+^* and *Ggt1^−/−^* females at the age of (N, O) 3 weeks, (P, Q) 5 weeks, and (R, S) 8 weeks. Histological sections were observed in the ovaries from (T, U) 3‐week‐old, and (V, W) 8‐week‐old *Ggt1^+/+^* and *Ggt1^−/−^* females stimulated with PMSG (*n* = 6 mice for each group). The relative mRNA and protein levels were normalized to those of β‐actin. Data are expressed as the mean ± SD from three independent experiments. *p < 0.05, **p < 0.01; N.S., none significant

**FIGURE 2:**
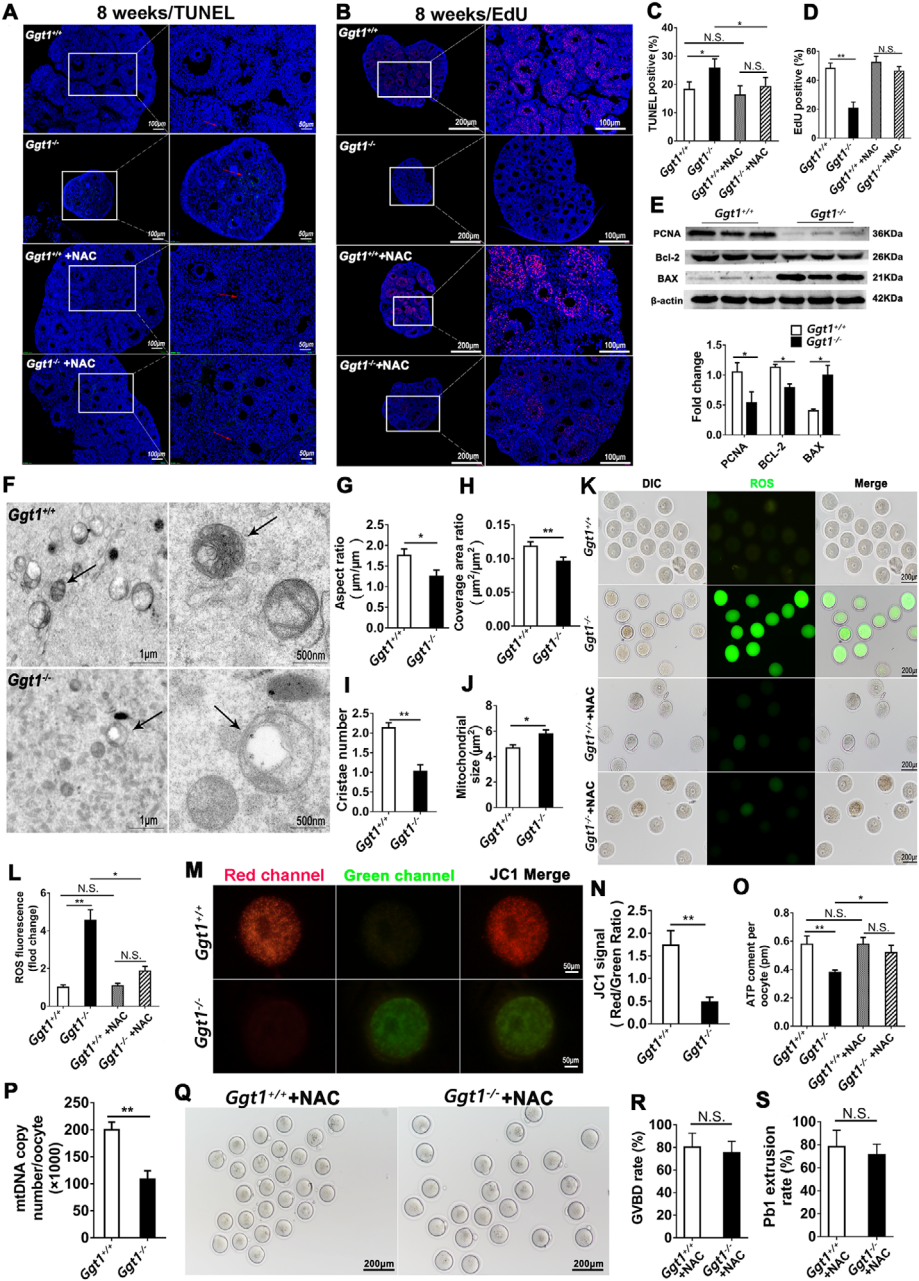
Granulosa cell growth, ovarian mitochondrial structure, and oocyte development in vitro are impaired in adult *Ggt1^−/−^* female mice. (A) Apoptosis of murine granulosa cells in 8‐week‐old *Ggt1^+/+^* and *Ggt1^−/−^* ovaries was evaluated by TUNEL assay. (B) EdU staining of ovaries from 8‐week‐old *Ggt1^+/+^* and *Ggt1^−/−^* mice (*n* = 6 mice for each group). The cell proliferation or apoptosis was indicated by arrows. The numbers of (C) TUNEL‐positive cells and (D) EdU‐positive cells in ovarian sections were shown in histograms. (E) Western blotting analysis of BAX, BCL2, and PCNA protein levels in *Ggt1^+/+^* and *Ggt1^−/−^* ovaries. The relative protein levels were normalized to those of β‐actin. (F) Representative electron microscopic photographs of the ovaries from 8‐week‐old *Ggt1^+/+^* and *Ggt1^−/−^* mice. Arrows show mitochondria. (G) The mitochondrial aspect ratio (mitochondrial length/width), (H) the mitochondrial coverage area ratio (mitochondrial area/cytoplasm area), (I) the mitochondrial cristae number, and (J) mitochondrial size were observed in *Ggt1^−/−^* ovaries compared to *Ggt1^+/+^* ovaries (*n* = 3 mice for each group). (K) Representative images of ROS fluorescence (green) in 8‐week‐old *Ggt1^+/+^* and *Ggt1^−/−^* oocytes. (L) Quantification of the relative ROS level. (M, N) Mitochondrial membrane potentials in 8‐week‐old *Ggt1^+/+^* and *Ggt1^−/−^* oocytes were assessed using JC‐1 staining. The fluorescent dye JC‐1 shifted from green to red with increasing ψ, indicating the compromised mitochondrial activity. (O) The ATP content per oocyte from 8‐week‐old *Ggt1^+/+^* and *Ggt1^−/−^* mice. (P) Quantitative analysis of mtDNA copy number in individual oocyte from 8‐week‐old *Ggt1^+/+^* and *Ggt1^−/−^* mice (*n* = 30 oocytes for each group). (Q) Representative images of the oocytes from *Ggt1^+/+^* and *Ggt1^−/−^* mice after 14 h of culture. Quantitative analyses of (R) germinal vesicle breakdown (GVBD) rate, and (S) first polar‐body (Pb1) extrusion rate were done in *Ggt1^+/+^* and *Ggt1^−/−^* oocytes (*n* = 200 oocytes for each group). (Q–S) Immature GV oocytes isolated from 8‐week‐old *Ggt1^+/+^* and *Ggt1^−/−^* mice treated with NAC were cultured in vitro to check their maturational progression. Data are expressed as the mean ± SD. *p < 0.05, **p < 0.01; N.S., none significant

We further checked the mitochondrial structure in *Ggt1^−/−^* ovaries by transmission electron microscopy. There were similar numbers and structures of the ovarian mitochondria in 3‐week‐old *Ggt1^−/−^* mice and *Ggt1^+/+^* mice (Figure S1C–G). Interestingly, ultra‐structural aberrations, especially vacuole formation and cristae loss were frequently observed in mitochondria of 8‐week‐old *Ggt1^−/−^* ovaries (Figure 2F–J). In addition, reactive oxygen species (ROS) level was increased, whereas mitochondrial membrane potential, ATP content, and mtDNA levels were remarkably reduced in *Ggt1^−/−^* oocytes (Figure 2K–P). Furthermore, Germinal vesicle (GV) oocytes derived from 3‐week‐old *Ggt1^−/−^* mice were unable to resume meiosis (Figure S1H). GV breakdown (GVBD) rate and the first polar‐body extrusion rate were dramatically decreased in *Ggt1^−/−^* oocytes compared with *Ggt1^+/+^* oocytes (Figure S1I,J). These results showed the reduced potential of development in *Ggt1^−/−^* oocytes. Furthermore, NAC was effective to restore *Ggt1^−/−^* mice growth retardation (Figure 1D), granulosa cell apoptosis (Figure 2A–D), mitochondrial dysfunction (Figure 2K,L, and O), and oocyte developmental defect (Figure 2Q–S).

Next, we used pig as an animal model to further explore the role of *GGT1* in folliculogenesis. Two independent cDNA libraries from pre‐ovulatory ovarian follicles in Large White (LW) and Meishan (MS) sows were sequenced with the high‐throughput Illumina Solexa system (Figure S2A,B). KEGG pathway analysis showed that the differentially expressed genes (DEGs) were enriched in multiple biological processes including metabolic activity, arachidonic acid metabolism, and steroid biosynthesis (Figure S2C,D). *GGT1* was involved in multiple biological processes such as arachidonic acid (AA) metabolism (Figure S2C). Porcine *GGT1* pre‐mRNA was subjected to alternative splicing analysis, generating a full‐length isoform (*GGT1‐01*) and an exon11 skipped isoform (*GGT1‐02*) (Figure 3A and Figure S2E,F). *GGT1‐02* which is equivalent to *Ggt1* in mice was highly expressed in MS ovaries (Figure S2G) and conserved in mammals (Figure S2H,I).

**FIGURE 3:**
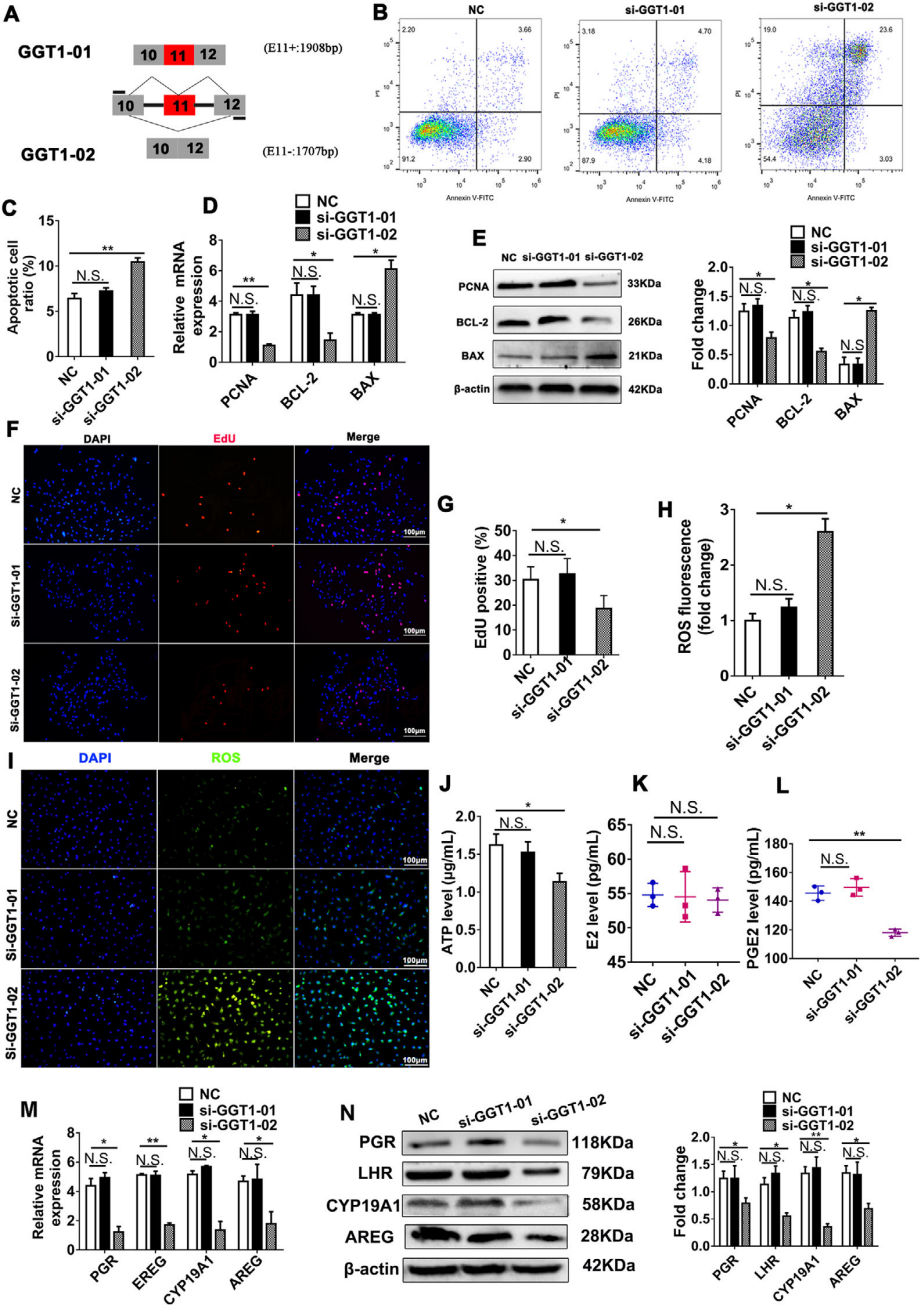
Porcine *GGT1‐02*, but not *GGT1‐01*, knockdown induces cell apoptosis, ROS accumulation, and decreases prostaglandin synthesis in porcine granulosa cells (pGCs). (A) Schematic diagram of *GGT1* variants including or lacking exon 11 (*GGT1‐01* and *GGT1‐02*). (B, C) Porcine granulosa cells were transfected with the NC, *si‐GGT1‐01*, and *si‐GGT1‐02* for 48 h, and cell apoptosis was assayed using flow cytometry. (D) qRT‐PCR and (E) Western blotting analysis of *BAX*, *BCL2*, and *PCNA* expression levels were carried out in pGCs. (F, G) pGC proliferation was assayed using EdU (proliferous cells are indicated in red). The cell nuclei are stained with DAPI (blue). (H) Quantification of the relative reactive oxygen species (ROS) level. (I) Representative images of ROS fluorescence in pGCs. (J) ATP content in pGCs. (K) Estrogen and (L) prostaglandin levels were assessed in pGCs. (M) qRT‐PCR and (N) Western blotting analysis of *PGR*, *EREG*, *CYP19A1*, and *EREG* expression were done in pGCs. (D–N) All samples were derived from pGCs transfected with NC, *si‐GGT1‐01*, and *si‐GGT1‐02*, respectively. The relative mRNA and protein levels were normalized to those of β‐actin. Data are expressed as the mean ± SD from three independent experiments. *p < 0.05, **p < 0.01; N.S., none significant

*GGT1‐02* knockdown significantly increased porcine ovarian granulosa cell (pGC) apoptosis and inhibited cell proliferation (Figure 3B–G). In *GGT1‐02* repressed pGCs, ROS level was increased, whereas ATP content, PGE2 synthesis, and the expression of follicular development‐related genes were decreased (Figure 3H–N). These results were further verified in pGCs with *GGT1‐02* overexpression (Figure S3A–J). However, *GGT1‐01* knockdown or overexpression had no significant effects on apoptosis and cell proliferation, ROS level, ATP content, estrogen and PGE2 synthesis, and follicular development‐related gene expressions (Figure 3B–N and Figure S3A–J).

GGT consists of one large subunit and one small subunit.^5^ Only when the large subunit and the small one form a complex, the GGT is highly active.^6^ The two subunits of GGT1‐02 were shown to directly interact with each other, but there was an unknown structure between the large and small subunits of GGT1‐01 (Figure S2E). ELISA results showed that GGT1‐02 had a higher GGT activity than GGT1‐01 (Figure 4A and Figure S4A,B). We found that GGT1‐02 regulated the Ca^2+^ stores, and promoted AA synthesis through activating the cPLA2 activity in pGCs (Figure 4B–K and Figure S4C–H). TMCO1 acts as a Ca^2+^ load‐activated calcium channel to release Ca^2+^ when there is an overload of ER Ca^2+^, thereby maintaining calcium homeostasis.^7^ The Co‐IP assay results verified that GGT1‐02 interacted with TMCO1, and the 949–1155 bp domain in GGT1‐02 was indispensable for PGE2 synthesis (Figure 4L–R). In addition, murine GGT1 interacted with TMCO1 to regulate PGE2 synthesis via the cPLA2‐AA‐PTGS2 pathway (Figure S5A–F).

**FIGURE 4:**
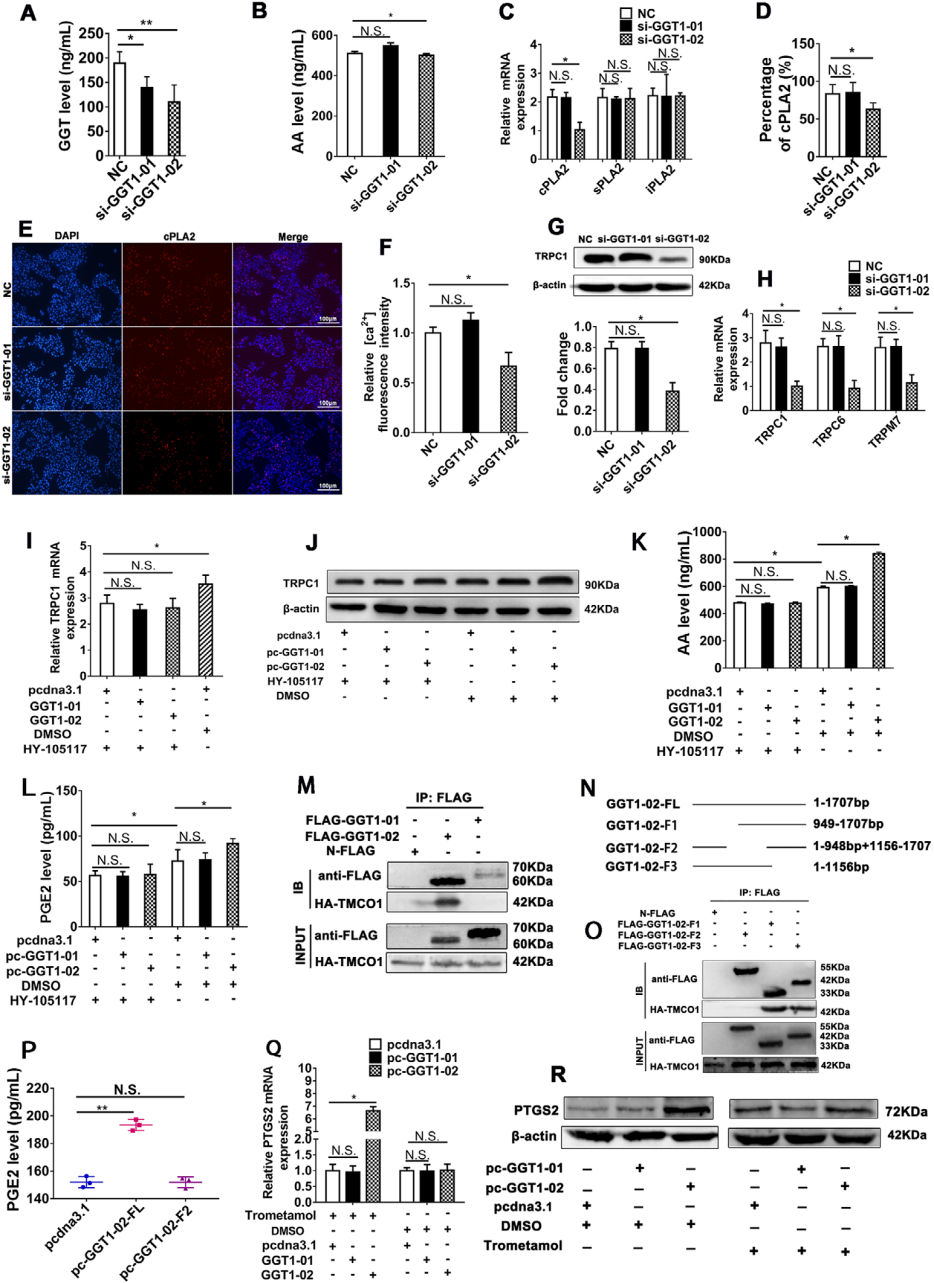
Porcine GGT1 variant 2 interacts with TMCO1 to regulate Ca^2+^ stores and promotes PGE2 synthesis through the cPLA2‐AA‐PTGS2 pathway. (A) The γ‐glutamyltransferase activity was tested using ELISA. (B) ELISA was used to examine the AA level in the culture media of porcine granulosa cells (pGCs). (C) qRT‐PCR and (D, E) immunofluorescence analysis were performed to identify the activity of major rate‐limiting enzymes in AA synthesis. (F) The relative Ca^2+^ levels in pGCs. (G) Western blotting and (H) qRT‐PCR analysis of *TRPC1* expression were done in pGCs. (A–H) All samples were derived from pGCs transfected with NC, *si‐GGT1‐01*, and *si‐GGT1‐02*, respectively. (I) qRT‐PCR and (J) Western blotting analysis of *TRPC1* expression were done in pGCs. ELISA was used to examine (K) AA and (L) PGE2 levels in the culture media of pGCs. (I–L) Porcine granulosa cells were treated with *pcdna3.1+*DMSO, *pcdna3.1+*HY‐105117, *pc‐GGT1‐01+*HY‐105117, and *pc‐GGT1‐02+*HY‐105117, respectively. HY‐10511 is one of the Ca^2+^ pathway inhibitors. (M) Immunoprecipitation assays using porcine kidney (PK) cells co‐transfected with *HA‐TMCO1*+*FLAG‐GGT1‐01* or *HA‐TMCO1*+*FLAG‐GGT1‐02*. (N) The truncated fragments of GGT1 (GGT1‐02‐F1, GGT1‐02‐F2, and GGT1‐02‐F3). (O) Co‐IP was used to examine the interaction between truncated fragments of GGT1 and TMCO1 in PK cells. (P) PGE2 level in pGCs overexpressing *GGT1‐02* or *GGT1‐02‐F2*. (Q) qRT‐PCR and (R) Western blotting were used to detect *PTGS2* expression levels in pGCs. (Q, R) Porcine granulosa cells were treated with trometamol or DMSO. Trometamol is one of the PTGS2‐PGE2 pathway inhibitors. The relative mRNA and protein levels were normalized to those of β‐actin. Data are expressed as the mean ± SD from three independent experiments. *p < 0.05, **p < 0.01; N.S., nonsignificant

In this study, we also explored the mechanism underlying porcine *GGT1* splicing. Our results demonstrated that both RNA recognition motif 2 (RRM2) and arginine/serine‐rich (RS) domains of SRSF1 were necessary for *GGT1* splicing (Figure S6A–H). RNA‐binding‐protein‐immunoprecipitation results indicated that SRSF1 is directly bound to the *GGT1* pre‐mRNA through the RRM domain (Figure S6I). The RS domain of serine/arginine‐rich (SR) protein is involved in protein‐protein interaction.^8^ Here, analysis on *pc‐SRSF1* co‐transfected with *GGT1*‐minigene carrying deletions of regulatory elements showed that *GGT1*‐minigene‐G4, which had a binding site of hnRNPH1, was a critical element for the SRSF1‐mediated *GGT1* splicing. SRSF1 interacted with hnRNPH1 and then promoted the production of the *GGT1‐02* transcript (Figure S6J–S). Meanwhile, SRSF1 mediated *GGT1* splicing to regulate porcine ovarian granulosa cell growth and PGE2 synthesis (Figure S7A–L).

In summary, *Ggt1* is required for ovarian follicular development, ovulation, and female fertility. *Ggt1* deletion generates mitochondrial dysfunction and ROS accumulation, leading to PCOS‐like phenotypes in female mice. NAC can alleviate mitochondrial dysfunction and oocyte developmental defect caused by *Ggt1* deficiency in female mice. GGT1 interacts with TMCO1 to activate the cPLA2 and accelerate PGE2 synthesis through the AA‐PTGS2‐PGE2 pathway in granulosa cells and promote follicular development (Figure S8). Our data demonstrate that NAC restores the PCOS‐like phenotypes in *Ggt1^−/−^* female mice, and extend the understanding of the potential therapeutic schedule associated with PCOS in human patients.

## CONFLICT OF INTEREST

The authors declare that there is no conflict of interest.

## ETHICS APPROVAL AND CONSENT TO PARTICIPATE

All animals received humane care according to the criteria outlined in the Guide for the Care and Use of Laboratory Animals. All animal experiments were conducted in accordance with the guidelines of the Animal Care and Ethics Committee of Huazhong Agricultural University.

## AUTHOR CONTRIBUTIONS

Fenge Li and L.W.conceived and designed the research. Ling Wang, Jinhua Tang, Jiawei Zhou, Lihua Zhu, Feng Tan, Yaru Chen, Lei Wang, Huibin Song, Shuqi Mei, and Yiliang Miao performed experiments. L.W. and Fenge Li analyzed the data. L.W. and Fenge Li wrote the manuscript. All authors read and approved the final manuscript.

## DATA AVAILABILITY STATEMENT

The data that support the findings of this study are available from Fenge Li upon reasonable request.

## Supporting information



Supporting information
**Figure S1 Granulosa cell growth, mitochondrial function, and oocyte development in vitro are observed in 3‐week‐old *Ggt1^−^^/−^* female mice. (A)** Apoptosis of granulosa cells in 3‐week‐old *Ggt1^+/+^* and *Ggt1^−/−^* ovaries was evaluated by TUNEL assay. **(B)** EdU staining of ovaries from 3‐week‐old *Ggt1^+/+^* and *Ggt1^−/−^* mice (n n = = 6 mice for each group). **(C)** Representative electron microscopic photographs of oocytes from 3‐week‐old *Ggt1^+/+^* and *Ggt1^−/−^* ovaries. The mitochondrial aspect ratio (mitochondrial length/width) **(D)**, the mitochondrial coverage area ratio (mitochondrial area/cytoplasm area) **(E),** the mitochondrial cristae number **(F)**, and mitochondrial size **(G)** were observed in *Ggt1^−/−^* ovaries compared to *Ggt1^+/+^* ovaries (n n = = 3 mice for each group). **(H)** Representative images of the oocytes from *Ggt1^+/+^* and *Ggt1^−/−^* mice after 14 h of culture. Quantitative analysis of the GVBD rate **(I)**, and Pb1 extrusion rate **(J)** in *Ggt1^+/+^* and *Ggt1^−/−^* oocytes (n n = = 200 oocytes for each group). **(H‐J)** Immature GV oocytes isolated from 3‐week‐old *Ggt1^+/+^* and *Ggt1^−/−^* mice were cultured *in vitro* to check their maturational progression. Data are expressed as the mean ± SD from three independent experiments. **P < < 0.01, N.S. none significant.

Supporting information
**Figure S2 Two splice variants of *GGT1* gene are identified by RNA‐seq in porcine ovaries. (A)** Volcano plot for the differentially expressed genes (DEGs) in the pre‐ovulatory ovarian follicles between MS and LW sows. **(B)** qRT‐PCR analysis was used to validate the differential expressions of *TPP1*, *HADH*, *EGR1*, *ATAR*, *ITGA2*, *CD151*, *CD44*, and *GGT1* genes. The relative mRNA levels were normalized to those of β‐actin. **(C)** KEGG pathway enrichment analysis of DEGs. **(D)** The heatmap depicting the expression profiles of 11 DEGs which are highly correlated with follicular development. **(E)** The three‐dimensional protein structure analysis of GGT1‐01 and GGT1‐02 proteins in pigs. I‐TASSER online server was used to predict the three‐dimensional structure of GGT1 variants. **(F)** RT‐PCR was used to detect *GGT1* variants in porcine ovaries. **(G)** qRT‐PCR was carried out to test XS% of *GGT1* in MS and LW ovaries. XS% % = = *GGT1‐02*/(*GGT1‐01*+*GGT1‐02*)**. (H)** The alignment of GGT1 protein in mouse, pig and human. The amino acid sequence is derived from NCBI database. **(I)** Structural homology analysis of *GGT1* gene in pig, human, and mouse. Data are expressed as the mean ± SD from three independent experiments. *P < < 0.05.

Supporting information
**Figure S3 Porcine *GGT1‐02*, but not *GGT1‐01*, overexpression restrains apoptosis and ROS accumulation, and increases prostaglandin synthesis in granulosa cells**. All samples were derived from pGCs transfected with *pcdna3.1*, *pc‐GGT1‐01*, and *pc‐GGT1‐02*, respectively. **(A)** Cell apoptosis was assayed by flow cytometry. qRT‐PCR **(B)** and Western blotting **(C)** of *BAX*, *BCL2* and *PCNA* expressions were analyzed in pGCs. **(D)** EdU staining was used to detect cell proliferation (red). **(E)** Quantification of the relative ROS level. **(F)** ATP content in pGCs. Estrogen **(G)** and prostaglandin **(H)** levels were assessed in pGCs. qRT‐PCR **(I)** and Western blotting **(J)** analysis of *PGR*, *LHR*, *CYP19A1* and *EREG* expressions were done in pGCs. The relative mRNA and protein levels were normalized to those of β‐actin. Data are expressed as the mean ± SD from three independent experiments. *P < < 0.05, **P < < 0.01, N.S. none significant.

Supporting information
**Figure S4 Porcine *GGT1‐02*, but not *GGT1‐01*, overexpression increases γ‐glutamyltransferase and AA levels in granulosa cells. (A)** Schematic representation of the regulatory elements on *GGT1‐01* (GGT1‐∆1 shows a small subunit deletion, and GGT1‐∆2 shows a large subunit deletion). γ‐glutamyltransferase **(B)** and AA **(C)** levels were assessed by ELISA in pGCs which were transfected with *pcdna3.1*, *pc‐GGT1‐01*, *pc‐GGT1‐02*, *pc‐GGT1‐∆1*, and *pc‐GGT1‐∆2*, respectively. qRT‐PCR **(D)** and immunofluorescence **(E)** were used to test the major rate‐limiting enzyme levels of AA synthesis in pGCs. **(F)** The relative Ca^2+^ levels in pGCs. qRT‐PCR **(G)** and Western blotting **(H)** analysis of *TRPC1* expression were done. **(D‐H)** All samples were derived from pGCs transfected with *pcdna3.1*, *pc‐GGT1‐01*, and *pc‐GGT1‐02*, respectively. The relative mRNA and protein levels were normalized to those of β‐actin. Data are expressed as the mean ± SD from three independent experiments. *P < < 0.05, **P < < 0.01, N.S. none significant.

Supporting information
**Figure S5 GGT1 interacts with TMCO1 to regulate PGE2 synthesis via the cPLA2‐AA‐PTGS2 pathway in mice. (A)** qRT‐PCR analysis of *Trpc1* expression in *Ggt1^+/+^* and *Ggt1^−/−^* ovaries. **(B)** The relative Ca^2+^ levels in *Ggt1^+/+^* and *Ggt1^−/−^* ovaries. qRT‐PCR **(C)** and Western blotting **(D)** analysis of *Trpc1*, *Ptgs2* and *Trpm7* expression levels were done in *Ggt1^+/+^* and *Ggt1^−/−^* ovaries. **(E)** Co‐IP was used to analyze interaction between GGT1 and TMCO1 in *Ggt1^+/+^* ovaries. **(F)** Co‐localization analysis of GGT1 and TMCO1 by immunofluorescence assay in *Ggt1^+/+^* ovaries. The relative mRNA and protein levels were normalized to those of β‐actin. Data are expressed as the mean ± SD from three independent experiments. *P < < 0.05, **P < < 0.01.

Supporting information
**Figure S6 SRSF1 is required for porcine *GGT1* alternative splicing. (A)** qRT‐PCR was performed for analyzing *GGT1* exon 11 inclusion/skipping levels (XS% % = = *GGT1‐02*/*GGT1‐01*+*GGT1‐02*). PK cells were transfected with *pcdna3.1*, *pc‐SRSF1*, *pc‐SRSF2*, *pc‐SRSF3*, *pc‐SRSF4*, *pc‐SRSF5*, and *pc‐SRSF6*. **(B)** qRT‐PCR assay was used to detect the *GGT1* XS% in PK cells transfected with NC or *si‐SRSF1*. **(C)** Schematic representation of the porcine *GGT1*‐minigene. The boxes represent the exons and the lines represent the introns. **(D)** *pc‐SRSF1* was co‐transfected with *GGT1*‐minigene in PK cells, and then qRT‐PCR assay was carried out to detect the XS% of *GGT1*. **(E)** qRT‐PCR assay was used to detect the *GGT1* XS% in PK cells co‐transfected with NC + *GGT1*‐minigene or *si‐SRSF1* + *GGT1*‐minigene. **(F)** Schematic representation of the SRSF1 and its variants lack of the RRM or RS domain. **(G)** Expressions of the variants were verified by immunoblotting with an anti‐HA antibody. **(H)** SRSF1 and its variants lack of the RRM or RS domain were co‐transfected into PK cells with *GGT1*‐minigene. qRT‐PCR was used to test *GGT1* XS%**. (I)** RNA‐binding‐protein‐immunoprecipitation (RIP) was used to test the interaction between SRSF1 and *GGT1* mRNA in PK cells. **(J)** Schematic representation of the regulatory elements on *GGT1* exon 11. **(K)** *GGT1*‐minigenes carrying individual mutations were transfected into PK cells treated with *pcdna3.1* or *pc‐SRSF1*. qRT‐PCR was carried out to detect the XS%. **(L)** *pc‐hnRNPH1* was transfected into PK cells treated with NC or *si‐SRSF1*. qRT‐PCR was used to detect the *GGT1* XS%. **(M)** Co‐IP was used to test interaction between SRSF1 and hnRNPH1 in PK cells. **(N)** qRT‐PCR assay was used to detect the *GGT1* XS% in PK cells which were transfected with *pcdna3.1* or *pc‐hnRNPH1*. **(O)** *pc‐hnRNPH1* was co‐transfected with *GGT1*‐minigene in PK cells, and qRT‐PCR assay was performed to analyze the *GGT1* XS%. **(P)** qRT‐PCR assay was used to detect the *GGT1* XS% in PK cells which were transfected with NC or *si‐hnRNPH1*. **(Q)** qRT‐PCR assay was carried out to test the *GGT1* XS% in PK cells which were transfected with *NC*+*GGT1*‐minigene or *si‐hnRNPH1*+*GGT1*‐minigene. **(R)** Schematic representation of the *HA‐hnRNPH1* and its variants lack of RRM or RS domain. **(S)** *HA‐hnRNPH1* and its variants lack of RRM or RS domain were co‐transfected into PK cells with *GGT1*‐minigene. qRT‐PCR was used to tested XS% of *GGT1*. The relative mRNA and protein levels were normalized to those of β‐actin. Data are expressed as the mean ± SD from three independent experiments. *P < < 0.05, **P < < 0.01, N.S. none significant.

Supporting information
**Figure S7 SRSF1 mediates porcine *GGT1* splicing, thus regulates cell survival and PGE2 synthesis in pGCs. (A and B)** MTT assay was used to detect pGC proliferation. **(C and D)** Annexin V‐FITC/PI and flow cytometry was carried out to detect pGC apoptosis. qRT‐PCR **(E and F)** and Western blotting **(G and H)** analysis of *SRSF1*, *PCNA*, *BCL‐2*, and *BAX* expression levels were done in pGCs. ELISA was used to examine AA **(I and J)** or PGE2 **(K and L)** levels in the culture media of pGCs. **(A, C, E, G, I, and K)** All samples were derived from pGCs transfected with *pcdna3.1* or *pc‐SRSF1*. **(B, D, F, H, J, and L)** All samples were derived from pGCs transfected with NC+*pcdna3.1*, *si‐SRSF1*+*pcdna3.1*, *si‐SRSF1*+*pc‐GGT1‐01*, and *si‐SRSF1*+*pc‐GGT1‐02*, respectively. The relative mRNA and protein levels were normalized to those of β‐actin. Data are expressed as the mean ± SD from three independent experiments. *P < < 0.05, **P < < 0.01, N.S. none significant.

Supporting information
**Figure S8 Schematic summary of the critical role of mitochondrial function, prostaglandin synthesis, and ovarian follicular development**. In summary, *Ggt1* is required for ovarian follicular development, ovulation, and female fertility. *Ggt1*‐deletion generates mitochondrial dysfunction and ROS accumulation, leading to PCOS‐like phenotypes in female mice; NAC can alleviate mitochondrial dysfunction and oocyte developmental defect caused by *Ggt1* deficiency in female mice; GGT1 interacts with TMCO1 to activate the cPLA2 and accelerate PGE2 synthesis through the AA‐PTGS2‐PGE2 pathway in granulosa cells, and promote follicular development.

Supporting information
