# Supplementary figures and images for: N‐Acetyl‐l‐cysteine restores reproductive defects caused by Ggt1 deletion in mice
Source: Clin Transl Med. 2021 Aug 25;11(8):e510. doi: 10.1002/ctm2.510 (PMC8387720; doi:10.1002/ctm2.510)

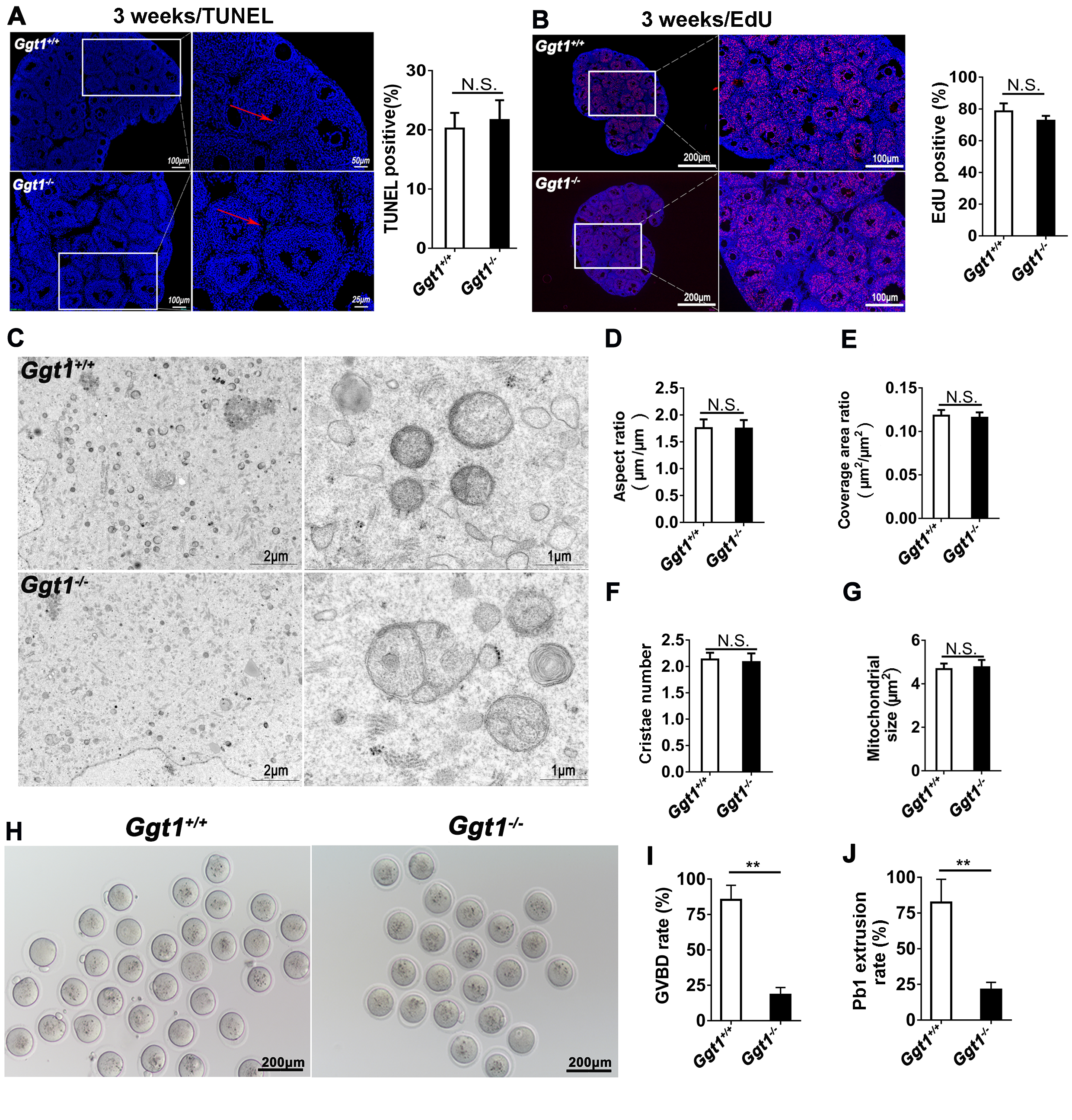

Supplement: Supplementary file 1 — Supporting information Figure S1 Granulosa cell growth, mitochondrial function, and oocyte development in vitro are observed in 3‐week‐old Ggt1− /− female mice. (A) Apoptosis of granulosa cells in 3‐week‐old Ggt1+/+ and Ggt1−/− ovaries was evaluated by TUNEL assay. (B) EdU staining of ovaries from 3‐week‐old Ggt1+/+ and Ggt1−/− mice (n n = = 6 mice for each group). (C) Representative electron microscopic photographs of oocytes from 3‐week‐old Ggt1+/+ and Ggt1−/− ovaries. The mitochondrial aspect ratio (mitochondrial length/width) (D), the mitochondrial coverage area ratio (mitochondrial area/cytoplasm area) (E), the mitochondrial cristae number (F), and mitochondrial size (G) were observed in Ggt1−/− ovaries compared to Ggt1+/+ ovaries (n n = = 3 mice for each group). (H) Representative images of the oocytes from Ggt1+/+ and Ggt1−/− mice after 14 h of culture. Quantitative analysis of the GVBD rate (I), and Pb1 extrusion rate (J) in Ggt1+/+ and Ggt1−/− oocytes (n n = = 200 oocytes for each group). (H‐J) Immature GV oocytes isolated from 3‐week‐old Ggt1+/+ and Ggt1−/− mice were cultured in vitro to check their maturational progression. Data are expressed as the mean ± SD from three independent experiments. **P < < 0.01, N.S. none significant. [file CTM2-11-e510-s009.tif]

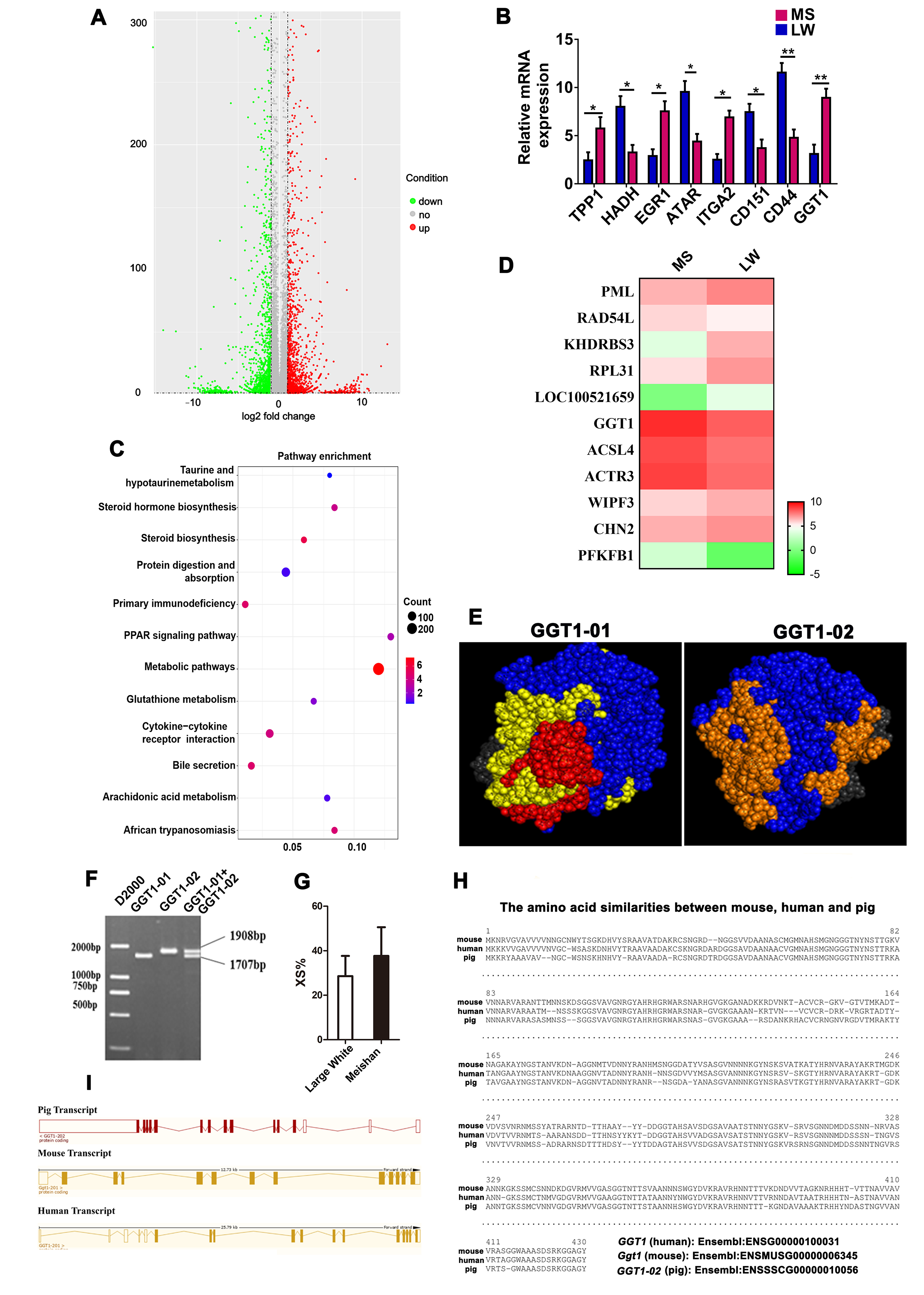

Supplement: Supplementary file 2 — Supporting information Figure S2 Two splice variants of GGT1 gene are identified by RNA‐seq in porcine ovaries. (A) Volcano plot for the differentially expressed genes (DEGs) in the pre‐ovulatory ovarian follicles between MS and LW sows. (B) qRT‐PCR analysis was used to validate the differential expressions of TPP1, HADH, EGR1, ATAR, ITGA2, CD151, CD44, and GGT1 genes. The relative mRNA levels were normalized to those of β‐actin. (C) KEGG pathway enrichment analysis of DEGs. (D) The heatmap depicting the expression profiles of 11 DEGs which are highly correlated with follicular development. (E) The three‐dimensional protein structure analysis of GGT1‐01 and GGT1‐02 proteins in pigs. I‐TASSER online server was used to predict the three‐dimensional structure of GGT1 variants. (F) RT‐PCR was used to detect GGT1 variants in porcine ovaries. (G) qRT‐PCR was carried out to test XS% of GGT1 in MS and LW ovaries. XS% % = = GGT1‐02/(GGT1‐01+GGT1‐02). (H) The alignment of GGT1 protein in mouse, pig and human. The amino acid sequence is derived from NCBI database. (I) Structural homology analysis of GGT1 gene in pig, human, and mouse. Data are expressed as the mean ± SD from three independent experiments. *P < < 0.05. [file CTM2-11-e510-s006.tif]

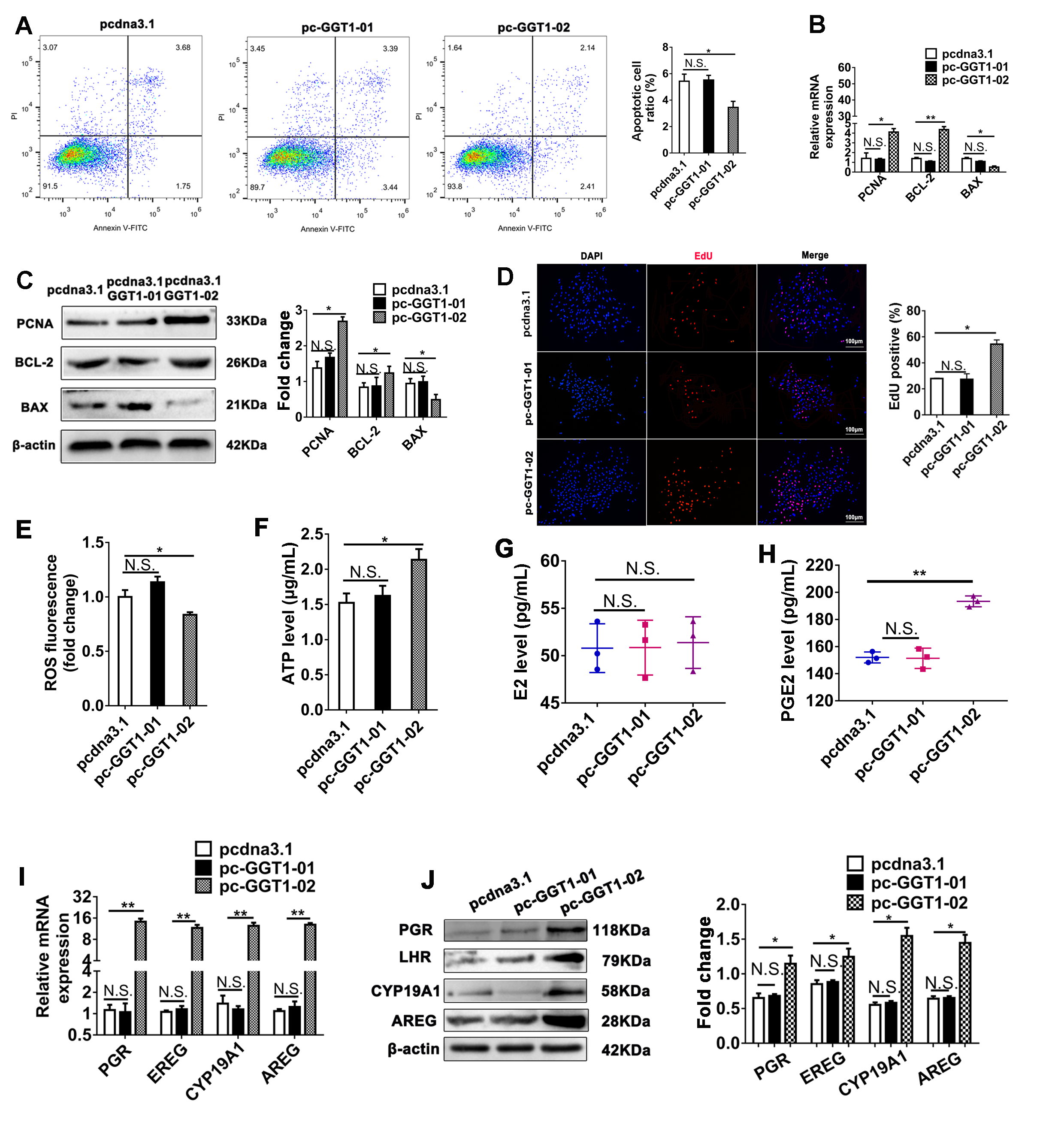

Supplement: Supplementary file 3 — Supporting information Figure S3 Porcine GGT1‐02, but not GGT1‐01, overexpression restrains apoptosis and ROS accumulation, and increases prostaglandin synthesis in granulosa cells. All samples were derived from pGCs transfected with pcdna3.1, pc‐GGT1‐01, and pc‐GGT1‐02, respectively. (A) Cell apoptosis was assayed by flow cytometry. qRT‐PCR (B) and Western blotting (C) of BAX, BCL2 and PCNA expressions were analyzed in pGCs. (D) EdU staining was used to detect cell proliferation (red). (E) Quantification of the relative ROS level. (F) ATP content in pGCs. Estrogen (G) and prostaglandin (H) levels were assessed in pGCs. qRT‐PCR (I) and Western blotting (J) analysis of PGR, LHR, CYP19A1 and EREG expressions were done in pGCs. The relative mRNA and protein levels were normalized to those of β‐actin. Data are expressed as the mean ± SD from three independent experiments. *P < < 0.05, **P < < 0.01, N.S. none significant. [file CTM2-11-e510-s002.tif]

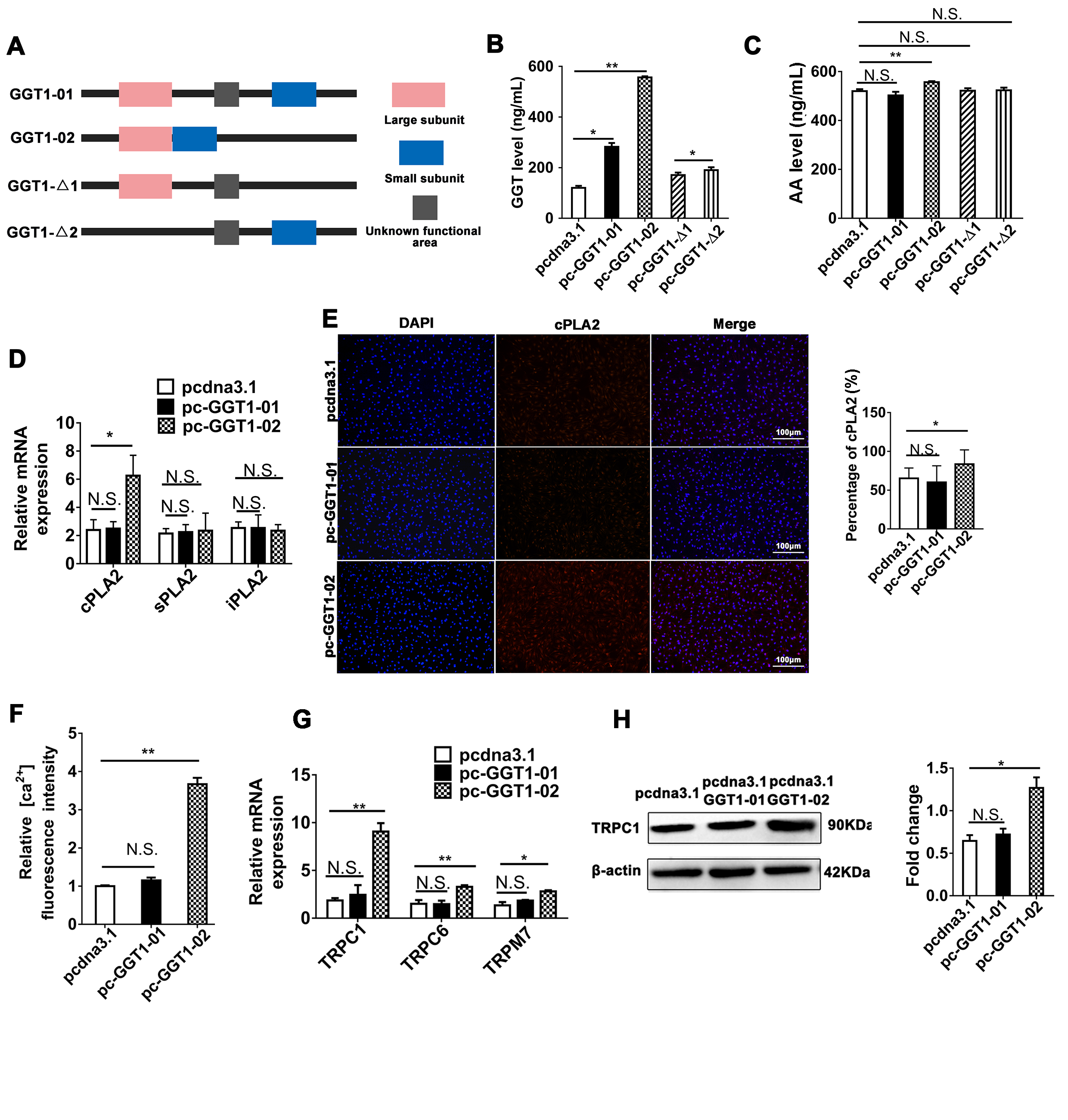

Supplement: Supplementary file 4 — Supporting information Figure S4 Porcine GGT1‐02, but not GGT1‐01, overexpression increases γ‐glutamyltransferase and AA levels in granulosa cells. (A) Schematic representation of the regulatory elements on GGT1‐01 (GGT1‐∆1 shows a small subunit deletion, and GGT1‐∆2 shows a large subunit deletion). γ‐glutamyltransferase (B) and AA (C) levels were assessed by ELISA in pGCs which were transfected with pcdna3.1, pc‐GGT1‐01, pc‐GGT1‐02, pc‐GGT1‐∆1, and pc‐GGT1‐∆2, respectively. qRT‐PCR (D) and immunofluorescence (E) were used to test the major rate‐limiting enzyme levels of AA synthesis in pGCs. (F) The relative Ca2+ levels in pGCs. qRT‐PCR (G) and Western blotting (H) analysis of TRPC1 expression were done. (D‐H) All samples were derived from pGCs transfected with pcdna3.1, pc‐GGT1‐01, and pc‐GGT1‐02, respectively. The relative mRNA and protein levels were normalized to those of β‐actin. Data are expressed as the mean ± SD from three independent experiments. *P < < 0.05, **P < < 0.01, N.S. none significant. [file CTM2-11-e510-s005.tif]

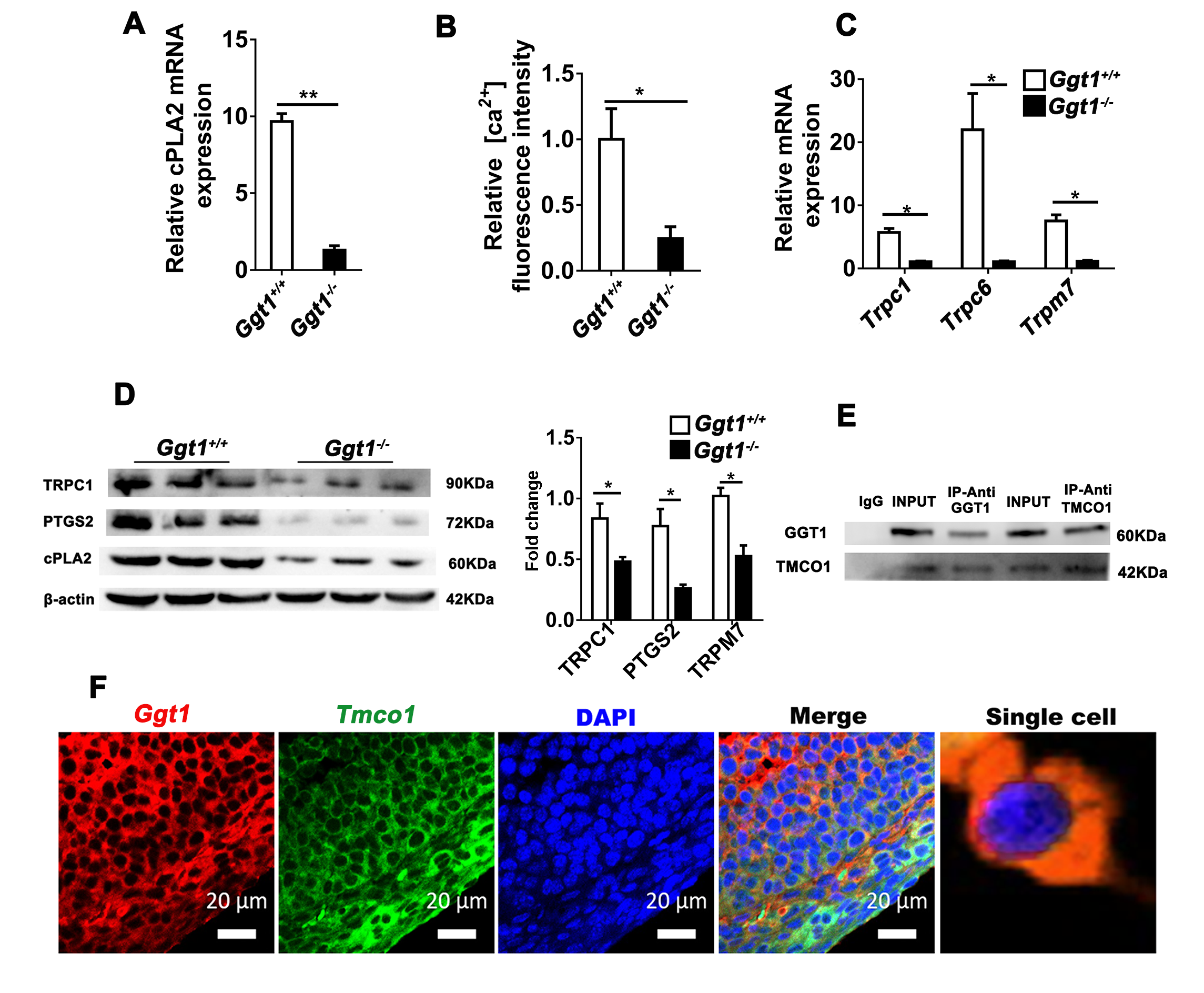

Supplement: Supplementary file 5 — Supporting information Figure S5 GGT1 interacts with TMCO1 to regulate PGE2 synthesis via the cPLA2‐AA‐PTGS2 pathway in mice. (A) qRT‐PCR analysis of Trpc1 expression in Ggt1+/+ and Ggt1−/− ovaries. (B) The relative Ca2+ levels in Ggt1+/+ and Ggt1−/− ovaries. qRT‐PCR (C) and Western blotting (D) analysis of Trpc1, Ptgs2 and Trpm7 expression levels were done in Ggt1+/+ and Ggt1−/− ovaries. (E) Co‐IP was used to analyze interaction between GGT1 and TMCO1 in Ggt1+/+ ovaries. (F) Co‐localization analysis of GGT1 and TMCO1 by immunofluorescence assay in Ggt1+/+ ovaries. The relative mRNA and protein levels were normalized to those of β‐actin. Data are expressed as the mean ± SD from three independent experiments. *P < < 0.05, **P < < 0.01. [file CTM2-11-e510-s008.tif]

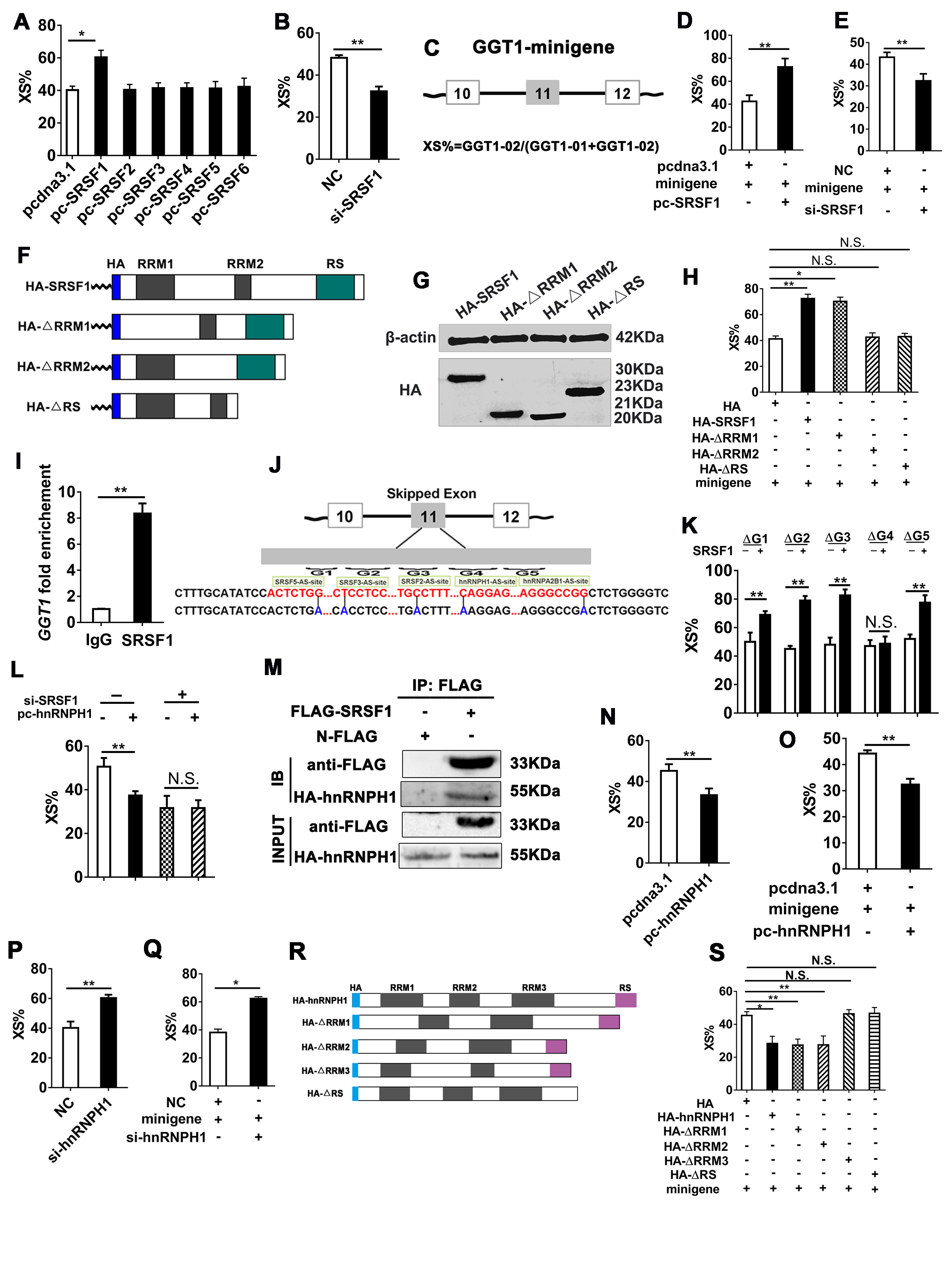

Supplement: Supplementary file 6 — Supporting information Figure S6 SRSF1 is required for porcine GGT1 alternative splicing. (A) qRT‐PCR was performed for analyzing GGT1 exon 11 inclusion/skipping levels (XS% % = = GGT1‐02/GGT1‐01+GGT1‐02). PK cells were transfected with pcdna3.1, pc‐SRSF1, pc‐SRSF2, pc‐SRSF3, pc‐SRSF4, pc‐SRSF5, and pc‐SRSF6. (B) qRT‐PCR assay was used to detect the GGT1 XS% in PK cells transfected with NC or si‐SRSF1. (C) Schematic representation of the porcine GGT1‐minigene. The boxes represent the exons and the lines represent the introns. (D) pc‐SRSF1 was co‐transfected with GGT1‐minigene in PK cells, and then qRT‐PCR assay was carried out to detect the XS% of GGT1. (E) qRT‐PCR assay was used to detect the GGT1 XS% in PK cells co‐transfected with NC + GGT1‐minigene or si‐SRSF1 + GGT1‐minigene. (F) Schematic representation of the SRSF1 and its variants lack of the RRM or RS domain. (G) Expressions of the variants were verified by immunoblotting with an anti‐HA antibody. (H) SRSF1 and its variants lack of the RRM or RS domain were co‐transfected into PK cells with GGT1‐minigene. qRT‐PCR was used to test GGT1 XS%. (I) RNA‐binding‐protein‐immunoprecipitation (RIP) was used to test the interaction between SRSF1 and GGT1 mRNA in PK cells. (J) Schematic representation of the regulatory elements on GGT1 exon 11. (K) GGT1‐minigenes carrying individual mutations were transfected into PK cells treated with pcdna3.1 or pc‐SRSF1. qRT‐PCR was carried out to detect the XS%. (L) pc‐hnRNPH1 was transfected into PK cells treated with NC or si‐SRSF1. qRT‐PCR was used to detect the GGT1 XS%. (M) Co‐IP was used to test interaction between SRSF1 and hnRNPH1 in PK cells. (N) qRT‐PCR assay was used to detect the GGT1 XS% in PK cells which were transfected with pcdna3.1 or pc‐hnRNPH1. (O) pc‐hnRNPH1 was co‐transfected with GGT1‐minigene in PK cells, and qRT‐PCR assay was performed to analyze the GGT1 XS%. (P) qRT‐PCR assay was used to detect the GGT1 XS% in PK cells which were transfected with NC or si‐ [file CTM2-11-e510-s007.tif]

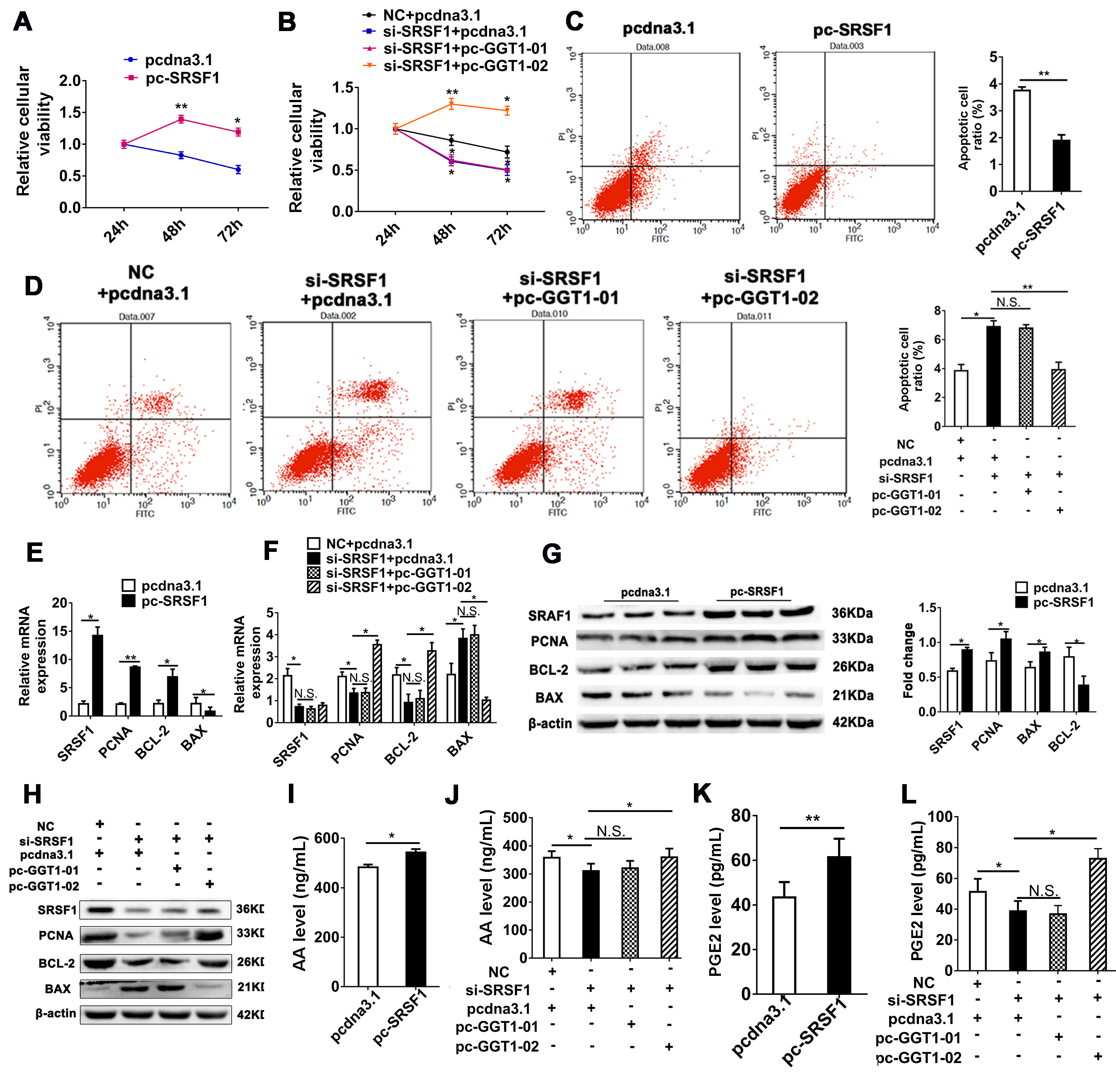

Supplement: Supplementary file 7 — Supporting information Figure S7 SRSF1 mediates porcine GGT1 splicing, thus regulates cell survival and PGE2 synthesis in pGCs. (A and B) MTT assay was used to detect pGC proliferation. (C and D) Annexin V‐FITC/PI and flow cytometry was carried out to detect pGC apoptosis. qRT‐PCR (E and F) and Western blotting (G and H) analysis of SRSF1, PCNA, BCL‐2, and BAX expression levels were done in pGCs. ELISA was used to examine AA (I and J) or PGE2 (K and L) levels in the culture media of pGCs. (A, C, E, G, I, and K) All samples were derived from pGCs transfected with pcdna3.1 or pc‐SRSF1. (B, D, F, H, J, and L) All samples were derived from pGCs transfected with NC+pcdna3.1, si‐SRSF1+pcdna3.1, si‐SRSF1+pc‐GGT1‐01, and si‐SRSF1+pc‐GGT1‐02, respectively. The relative mRNA and protein levels were normalized to those of β‐actin. Data are expressed as the mean ± SD from three independent experiments. *P < < 0.05, **P < < 0.01, N.S. none significant. [file CTM2-11-e510-s004.tif]

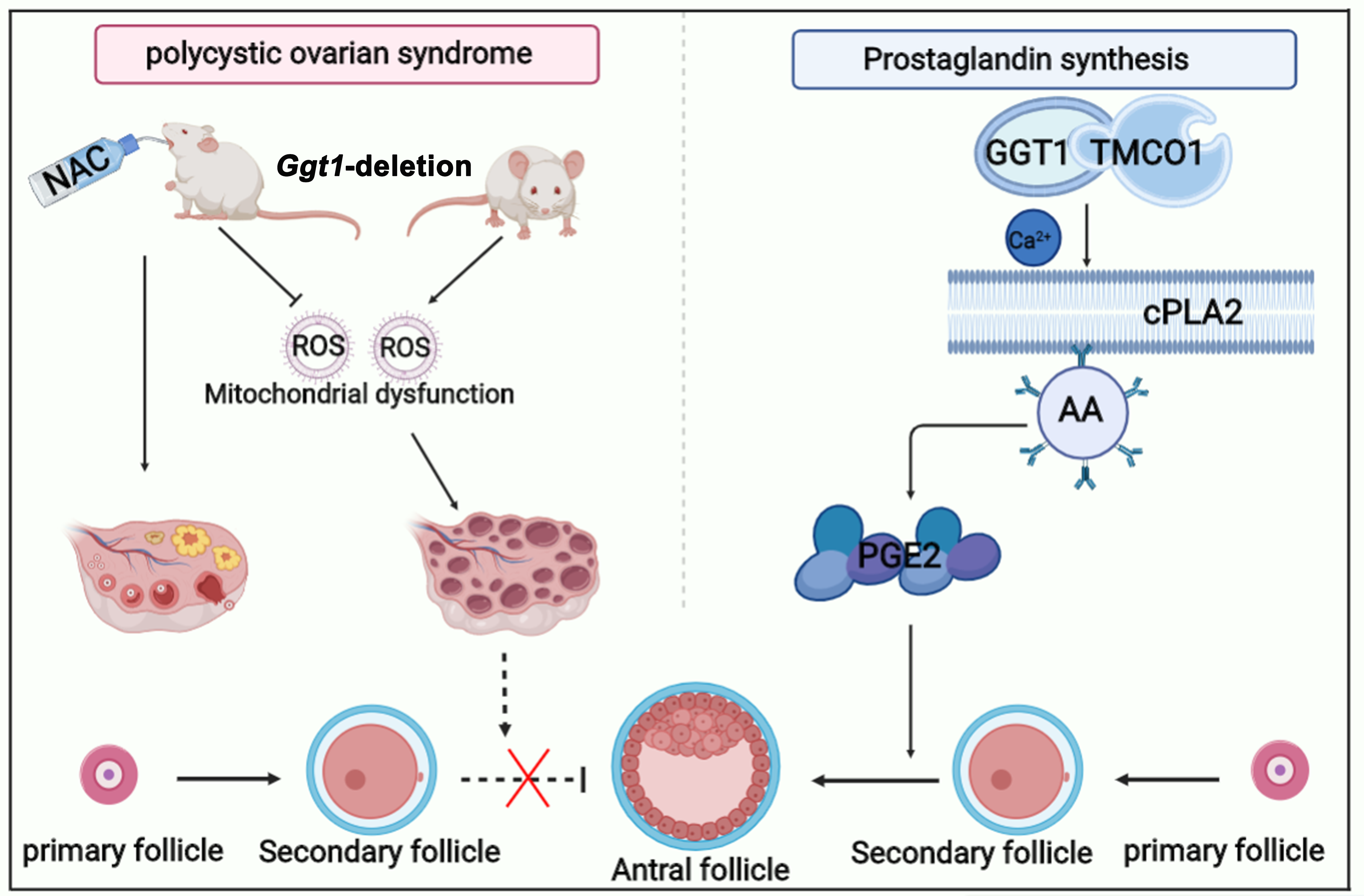

Supplement: Supplementary file 8 — Supporting information Figure S8 Schematic summary of the critical role of mitochondrial function, prostaglandin synthesis, and ovarian follicular development. In summary, Ggt1 is required for ovarian follicular development, ovulation, and female fertility. Ggt1‐deletion generates mitochondrial dysfunction and ROS accumulation, leading to PCOS‐like phenotypes in female mice; NAC can alleviate mitochondrial dysfunction and oocyte developmental defect caused by Ggt1 deficiency in female mice; GGT1 interacts with TMCO1 to activate the cPLA2 and accelerate PGE2 synthesis through the AA‐PTGS2‐PGE2 pathway in granulosa cells, and promote follicular development. [file CTM2-11-e510-s003.tif]
