# Supplementary material for: N‐Acetyl‐l‐cysteine restores reproductive defects caused by Ggt1 deletion in mice
Source: Clin Transl Med. 2021 Aug 25;11(8):e510. doi: 10.1002/ctm2.510 (PMC8387720; doi:10.1002/ctm2.510)
Supplement: Supplementary file 9 — Supporting information [file CTM2-11-e510-s001.docx]

**Materials**

**Generation of *Ggt1* deficient mice**

*Ggt1* KO mice were generated using the CRISPR genome editing system with the BDF1 background according to a previous report.^1^ Two single guide RNAs (sgRNAs) targeting exon 2 of *Ggt1* gene in mouse genome were designed using an online CRISPR design tool (http://tools.geneome-engineering.org), inserted into the *px459* vector (48139, Addgene, USA). The 33 bp genomic region of *Ggt1* transcript was deleted using these sgRNAs (Table S1).

**NAC supplementation for weanling mice**

Three-week-old female mice (breastfeeding before the experiment) were divided randomly into two groups (n = 10/group): (i) mice received a basal diet and normal drinking water; (ii) mice received a basal diet and drinking water supplemented with NAC (HY-B0215, MedChemExpress, China) at a dosage of 10 mg/mL.^2^ After 5 weeks of NAC addition, mice were sacrificed.

**Enzyme-linked immunosorbent assay (ELISA)**

The *Ggt1^+/+^* and *Ggt1^-/-^* female mice aged around 8 weeks were killed at the proestrus stage.^3^ Blood was collected from an eyeball. The serum was separated and stored at -80 °C until use. Levels of serum FSH (RD-FSH-Mu, Enzo Life Sciences, USA), LH (EM1188, Enzo Life Sciences, USA), E2 (LS-F21360, Enzo Life Sciences, USA), PGE2 (OKEH02544, Enzo Life Sciences, USA), and testosterone (LS-F28645, Enzo Life Sciences, USA) were measured using ELISA kit. Porcine ovarian granulosa cells (pGCs) were cultured in a six-well plate with 2 mL medium, and transfected with oligonucleotides or plasmids; after 48 h, cell media were collected to measure E2 and PGE2 levels using an ELISA kit. Normalized hormone values were determined by extrapolating from a standard curve.

**Superovulation and oocyte collection**

Female mice at 23 days of age were killed by cervical dislocation at 48 h after injection with pregnant mare serum gonadotrophin (PMSG, 7 IU). Ovaries were dissected and repeatedly punctured in M2 medium (M7167, Sigma, USA). Cumulus-oocyte complexes were released from antral follicles. GV stage oocytes were collected, and then cultured in M16 medium (M7292, Sigma, USA) covered by mineral oil (M8410, Sigma, USA). To avoid spontaneous maturation of GV oocytes, 0.1 mg/mL dbcAMP (D2060, Sigma, USA) was added in the medium.

**Histology hematoxylin-eosin (H&E) staining**

Ovaries from *Ggt1^+/+^* and *Ggt1^-/-^* mice at 3, 5 and 8 weeks of age were fixed with 4% paraformaldehyde overnight, dehydrated in ethanol, embedded in paraffin, and sectioned at 5 μm. The sections were stained with hematoxylin and eosin for histological examination. Primary follicles, secondary follicles, antral follicles and [atretic](javascript:;) follicles were classified as described previously.^4^

**Cell proliferation assays using** **EdU staining and MTT assays**

Cell proliferation was measured by EdU staining and MTT assays. EdU staining was conducted using the BeyoClick ™ EdU Cell Proliferation Kit (ST067, Beyotime, China) according to the manufacturer’s instructions. The cells were cultured in DMEM supplemented with EdU and 20% FBS for 2 h, at 37 °C and 5% CO_2_. The *Ggt1^-/-^* and *Ggt1^+/+^* mice were injected with EdU (100 mg/kg) and sacrificed at 6 h post-injection. MTT (G4101-1000T, Servicebio, China) analysis was performed,^5^ to detect cell viability by measuring the optical density at 570 nm at 24 h, 48 h, and 72 h after transfection.

**TUNEL assay**

Ovaries were fixed in paraformaldehyde, embedded in paraffin and sectioned (5 μm). TUNEL staining was performed using In Situ Cell Death Detection kit, Fluorescein (G1501, Servicebio, China). Briefly, ovarian paraffin sections were dewaxed, rehydrated, then immersed in Tris-HCl (0.1 mol/L, pH 7.5) containing 3% BSA and 20% fetal bovine serum, and incubated with the TUNEL reaction Mixture for 1 h at 37 ℃. Images were obtained with microscope (Olympus, Japan).

**Transmission electron microscopy**

We processed the samples as described previously to analyze the mitochondrial structure.^6^ Briefly, the paracentral tissue of mouse ovaries was cut into 1mm piece, fixed in 2.5% glutaraldehyde and 1% osmic acid, dehydrated, and then incubated in the embedding medium overnight. Sections were made by ultra-microtome, and then analyzed under an electron transmission microscope after a lead citrate staining.

**ROS evaluation**

We used DCFH-DA (D6883, Sigma, USA) to detect the ROS level in living oocytes. DCFH-DA was prepared in DMSO prior to loading. Oocytes were incubated with 10 μmol/L DCFH-DA for 30 min at 37 °C, and then immediately observed under laser confocal scanning microscopy.

**Determination of** **mtDNA copy number and ATP content**

The mtDNA extraction and qRT-PCR were conducted as reported previously.^6^ Briefly, 50 oocytes were loaded in a PCR tube with 20 µL lysis buffer and processed for qRT-PCR analysis. Five 10-fold serial dilutions of purified plasmid standard DNAs were used to generate the standard curve. Mouse mtDNA-specific primers are listed in Table S1. Total ATP content was determined using the bioluminescent somatic cell assay kit (A6559, Sigma, USA) as described previously.^7^ Briefly, a 6‐point standard curve (0, 0.1, 0.5, 1.0, 10, and 50 pmol/L of ATP) was generated in each assay, and the ATP content was calculated by using the formula derived from the linear regression of the standard curve.

**Solexa sequencing and bioinformatical analysis**

Three multiparous MS sows and 3 multiparous LW sows that exhibited normal estrous cycles were raised on the pig farm of Huazhong Agricultural University. The sows were treated with 1000 IU PMSG and 500 IU hCG as previously described.^8^ Solexa sequencing was carried out at the Beijing Genomics Institute (BGI). Raw reads were filtered into clean reads which were then aligned to the reference swine genome Sscrofa10.2 with SOAPaligner/SOAP2.

Differentially expressed genes between MS and LW ovarian follicles were identified with an R package named DEGseq. The genes with false discovery rate (FDR) ≤0.001 and |log2Ratio|≥1 were taken as DEGs. We mapped all differentially expressed genes (DEGs) to GO terms in the database (<http://www.geneontology.org/>). KEGG (<http://www.genome.jp/kegg/pathway.html>) was used to perform pathway enrichment analysis of DEGs.

**Quantitative real-time PCR (qRT-PCR)**

Total RNA was extracted using the TRIzol Reagent (15596026, Thermo Fisher Scientific, USA), and treated with RNase-free DNase (M610A, Promega, USA). Synthesis of cDNA was performed using 1 μg total RNA from each sample using RevertAid RT Reverse Transcription Kit (K1691, Thermo Fisher Scientific, USA). Quantitative real-time PCR was performed using the iTaq TM Universal SYBR Green Super Mix (172-5121, Bio-Rad, USA) and analyzed using CFX384 Touch™ Real-Time PCR Detection System (Bio-Rad, USA). Primers used in the qRT-PCR are shown in Table S1. Gene expression levels were normalized to the expression of *β-actin* using Gene Expression Macro software (Bio-Rad, USA) by using the 2 ^−ΔΔCt^ method.^9^

**Cell culture and cell transfection**

Porcine granulosa cells were isolated from porcine ovaries collected from a local slaughterhouse in Wuhan. PK-15 cells were obtained from the China Centre for Type Culture Collection (GDC061, Shanghai, China). Cells were cultured in (DMEM)/F-12 (11320033, Gibco, USA or SH30256.01, HyClone, USA) supplemented with 10% fetal bovine serum (10099141C, Gibco, USA), 100 U/mL penicillin and 100 mg/mL streptomycin (15140122, Gibco, USA) at 37 °C in a humidified atmosphere of 5% CO_2_. Cells were incubated in plates and grew up to 70% confluency at the time of transfection. Plasmids or siRNAs were transfected into the cells using Lipofectamine™ 3000 (L3000015, Thermo Fisher Scientific, USA) or RNAiMAX transfection reagent (13778030, Thermo Fisher Scientific, USA), respectively.

**Western blotting**

Western blotting analysis was performed following the standard procedures.^10^ Briefly, protein lysates were generated using RIPA Lysis Buffer (P0013B, Beyotime, China) with 1% Protease inhibitor cocktail (HY-K0010T, MCE, China), 1% Phosphatase Inhibitor Cocktail I (HY-K0021, MCE, China) and 1% Phosphatase Inhibitor Cocktail II (HY-K0022, MCE, China). Protein extracts were separated in 10% SDS-polyacrylamide gels and then transferred onto 0.22 μm PVDF membranes (iseq00010, Millipore, USA). The membranes were blocked with 5% non-fat dried milk or 3% BSA in TBST (20 mmol/L Tris-HCl, pH 7.5, 150 mmol/L NaCl, 0.1% Tween-20), and then incubated with a specific antibody listed in Table S2. The ECL kit (170-5061, Bio-Rad, USA) was used to detect immunoreactive protein bands by ChemiDoc MP Imaging System (Bio-Rad, USA). Image J was used to quantify the signal.

**Immunofluorescence staining**

Ovaries or cells were fixed in 4% PFA for 10-30 min, permeabilized in 0.1% Triton X-100 for approximately 10 min, blocked in 10% goat serum or 5% BSA in PBS (10 mmol/L sodium phosphate, 0.15M NaCl, pH 7.4 at 22 ℃). Thereafter, samples were incubated with a specific primary and the corresponding secondary antibody listed in Table S2, and co-stained with 4’, 6-diamidino-2-phenylindole (DAPI) (D9542-1M, Sigma, USA) to visualize cell nuclei.

**Small interfering RNA** **and plasmid construction**

Small interfering RNAs (siRNAs) listed in Table S1 were sythenized by GenePharma (Suzhou, China). The full-length coding sequences (CDSs) were amplified and separately cloned into *pcDNA3.1(+)* vector (D2951, Beyotime, China), *pCMV-FLAG* (D2632, Beyotime, China) and *pCMV-HA* (D2639, Beyotime, China). *GGT1*-minigene was amplified from genomic DNAs and then cloned into the *pcDNA3.1(+)* vector. Site-directed mutants from *GGT1* minigene vector were generated using overlap-extension PCR.

**Co-immunoprecipitation (Co-IP)**

The extracted proteins were incubated with 3 μg target antibodies overnight at 4 ℃. Next, 50 μL of Protein A/G magnetic beads (10006D, Bio-Rad, USA) was added to each incubation sample for 1 h at room temperature. The beads were washed three times with 1×PBS. Finally, the co-immunoprecipitated proteins were eluted by 1×SDS buffer, heated for 10 min at 70 ℃, separated on 10% SDS-polyacrylamide gels, transferred onto PVDF membranes for the immune-blot analysis. The related antibodies are listed in Table S2.

**RNA binding protein immunoprecipitation Assay (RIP)**

RIP was performed using the RNA-Binding Protein Immunoprecipitation Kit (17-700, Millipore, USA) according to the manufacturer’s instructions. The SRSF1 antibody (ab133689, Abcam, UK) was used for RIP. Co-precipitated RNAs were detected using qRT-PCR.

**Sequence analysis**

The SFmap software (http://sfmap.technion.ac.il/index.htmL) was used to predict the splicing factors in the *GGT1* skipped exon 11. I-TASSER (https://zhanglab.ccmb.med.umich.edu/I-TASSER/) was used to predict the three-dimensional structure of GGT1 variants.

**References**

**1.** Wang H, Yang H, Shivalila CS, et al. One-step generation of mice carrying mutations in multiple genes by CRISPR/Cas-mediated genome engineering. *Cell***.** 2013; 153(4):910-918.

**2.** Lieberman MW, Wiseman AL, Shi ZZ, et al. Growth retardation and cysteine deficiency in gamma-glutamyl transpeptidase-deficient mice. *Proc Natl Acad Sci U S A***.** 1996; 93(15):7923-7926.

**3.** Sun Z, Zhang H, Wang X, et al. TMCO1 is essential for ovarian follicle development by regulating ER Ca(2+) store of granulosa cells. *Cell Death Differ***.** 2018; 25(9):1686-1701.

**4.** Guzeloglu-Kayisli O, Lalioti MD, Aydiner F, et al. Embryonic poly(A)-binding protein (EPAB) is required for oocyte maturation and female fertility in mice. *Biochem J***.** 2012; 446(1):47-58.

**5.** Chen X, He L, Zhao Y, et al. Malat1 regulates myogenic differentiation and muscle regeneration through modulating MyoD transcriptional activity. *Cell Discov***.** 2017; 3:17002.

**6.** Wang Q, Ratchford AM, Chi MM, et al. Maternal diabetes causes mitochondrial dysfunction and meiotic defects in murine oocytes. *Mol Endocrinol***.** 2009; 23(10): 1603-1612.

**7.** Hou X, Zhang L, Han L, et al. Differing roles of pyruvate dehydrogenase kinases during mouse oocyte maturation. *J Cell Sci***.** 2015; 128(13):2319-2329.

**8.** Torner H, Brüssow KP, Alm H, Ràtky J, Kanitz W. Morphology of porcine cumulus-oocyte-complexes depends on the stage of preovulatory maturation. *Theriogenology***.** 1998; 50(1):39-48.

**9.** Livak KJ, Schmittgen TD. Analysis of relative gene expression data using real-time quantitative PCR and the 2(-Delta Delta C(T)) Method. *Methods***.** 2001; 25(4):402-408.

**10.** Sheng Y, Song Y, Li Z, et al. RAB37 interacts directly with ATG5 and promotes autophagosome formation via regulating ATG5-12-16 complex assembly. *Cell Death Differ***.** 2018; 25(5):918-934.

**Table S1**. **The oligonucleotide sequences**

| **Name** | **Sequence (5’-3’)** |
| --- | --- |
| mmu-*Ggt1*-GT-PF | AGTGAAGGTAGGATCCAGAGCT |
| mmu-*Ggt1*-GT-PR | AGAGAGACACGCTGAAAAGAGG |
| ssc-pcdan3.1-*GGT1*-PF | CTAGCTAGCGCCACCATGAAGAAGCGGTACCTGCT |
| ssc-pcdan3.1-*GGT1*-PR | CCAAGCTTTCATCAGTAGCCTGCAGGCTCCC |
| ssc-HA-GGT1-PF | TAGCCCGGGCGGATCCAAGCTTATGAAGAAGCGGTACCTGC |
| ssc-HA-GGT1-PR | GGCCCACTAGTTCTAGACTCGAGAGATCTTCAGTAGCCTGCA |
| ssc-pc-*PTGS2*-PF | CTAGCTAGCGCCACCATGGCTTACCCTTTCCAACT |
| ssc-pc-*PTGS2*-PR | CCAAGCTTAACCTGTTAATATTGATGTT |
| ssc-pc-*SRSF1*-PF | CTAGCTAGCGCCACCATGTCGGGAGGTGGTGTGAT |
| ssc-pc-*SRSF1*-PR | CTCTAGACTCGAGCGGCCGCTTATGTACGAGAGCGAG |
| ssc-pc-*SRSF2*-PF | CTA GCTAGCATGAGCTACGGCCGCCCGCC |
| ssc-pc-SRSF2-PF | CTCTAGACTCGAGCGGCCGCTTAAGAGGACACCGCTCCTT |
| ssc-pc-*SRSF3*-PF | CTA GCTAGCATGCATCGTGATTCCTGTCC |
| ssc-pc-SRSF3-PF | CTCTAGACTCGAGCGGCCGCCTATTTCCTTTCATTTGACC |
| ssc-pc-*SRSF4*-PF | CTA GCTAGCATGCCGCGGGTGTACATCGG |
| ssc-pc-SRSF4-PF | CTCTAGACTCGAGCGGCCGCTTAGGACCTTGAGTGGGACC |
| ssc-pc-*SRSF5*-PF | CTA GCTAGCATGAGTGGCTGTCGAGTATT |
| ssc-pc-SRSF5-PF | CTCTAGACTCGAGCGGCCGCTTAATTGCCACTGTCAACTG |
| ssc-pc-*SRSF6*-PF | CTA GCTAGCATGCCGCGCGTCTACATAGG |
| ssc-pc-SRSF6-PF | CTCTAGACTCGAGCGGCCGCTTAATCCCTGGAACTCGATC |
| ssc-pc-*hnRNPH1*-PF | CTAGCTAGCACGCCCGAACGCACTA |
| ssc-pc-*hnRNPH1*-PR | CCAAGCTTAAACAATCCTGATTCCCACTT |
| ssc-*GGT1*-minigene-PF | CCCAAGCTTGCCCCCATCTCAAGGTAGCCAAGT |
| ssc-*GGT1*-minigene-PR | CGGAATTCATCCAGCAAATCTGAATACATCC |
| ssc-minigene-G1-PF | CTTTGCATATCCACTCTGACCTTATGTGAGGTGCCTCTTCACATGTCACCTC |
| ssc-minigene-G1-PR | CTCACATAAGGTCAGAGTGGATATGCAAAGGCCCTGAGGCATTCCTCG |
| ssc-minigene-G2-PF | TTCACATGTCACCACCTCCAAGAAGCCCCCCTTGACGACAACACCCTCA |
| ssc-minigene-G2-PR | GGGGCTTCTTGGAGGTGGTGACATGTGAAGAGGCACCTCACATAAGGCCA |
| ssc-minigene-G3-PF | ACATCCCTGTCTGACTTTGTCTTCCCTGCCAGGAGGGCCGG |
| ssc-minigene-G3-PR | CAGGGAAGACAAAGTCAGACAGGGATGTGCTGGGAAGAACAAGCCAG |
| ssc-minigene-G4-PF | TCTTCCCTGCTAGGAGGGCCGGCTCTGGGGTCTCGGCA |
| ssc-minigene-G4-PR | GAGCCGGCCCTCCTAGCAGGGAAGACAAAGGCAGACAGGGAT |
| ssc-minigene-G5-PF | CCTGCCAGGAGGGCCGACTCTGGGGTCTCGGCAGGTGGTCC |
| ssc-minigene-G5-PR | GACCCCAGAGTCGGCCCTCCTGGCAGGGAAGACAAAGGCAGACAGGG |
| ssc-FLAG-TMCO1- PF | TAGCCCGGGCGGATCCAAGCTTATGAGCACCATGTTCGCGGA |
| ssc-FLAG-TMCO1- PR | GTACCGGGCCCACTAGTTCTAGACTCGAGTCAAGAGAACTTCCCAGAAG |
| ssc-*GGT1*-qRT-PCR-PF | AGCATGGGCATCGGGGGT |
| ssc-*GGT1*-qRT-PCR-PR | ATGGCGTCCTGGCTTCTC |
| ssc*-GGT1-01*-qRT-PCR-PF | GACCCCAAGTTTGTCAATGTG |
| ssc-*GGT1-01*-qRT-PCR-PR | GTGAGTGGTGGTATCGGAGA |
| ssc-*GGT1-02*-qRT-PCR-PF | ATGTGACCGAGGTGGTCC |
| ssc-*GGT1-02*-qRT-PCR-PR | AGTAGAGGTTGATGGTGCTGGTGGC |
| ssc-*SRSF1*-qRT-PCR-PF | CGACGGCTATGATTACGATG |
| ssc-*SRSF1*-qRT-PCR-PR | TTCAGAACGCCTGGATGG |
| ssc*-hnRNPH1*-qRT-PCR-PF | CGTTGAAATGGATTGGGTGTT |
| ssc-*hnRNPH1*-qRT-PCR-PR | TGTGCCCTATTCTTTCCTTGTG |
| ssc-*PCNA*-qRT-PCR-PF | ACCGCTGCGACCGCAATTTG |
| ssc*-PCNA*-qRT-PCR-PR | ACGTGCAAATTCACCAGAAGGCATC |
| ssc-*BCL-2*-qRT-PCR-PF | CAGGGTATGATAACCGGG |
| ssc-*BCL-2*-qRT-PCR-PR | CTGGACATCTCGGCAAAGTCG |
| ssc-*BAX*-qRT-PCR-PF | CCGAAATGTTTGCTGACG |
| ssc-*BAX*-qRT-PCR-PR | AGCCGATCTCGAAGGAAGT |
| mmu-*Pcna*-qRT-PCR-PF | GAAGAAGGTGCTGGAGG |
| mmu-*Pcna*-qRT-PCR-PR | TTTGGACATGCTGGTGA |
| mmu-*Bcl-2*-qRT-PCR-PF | GCTACCGTCGTGACTTCGC |
| mmu-*Bcl-2*-qRT-PCR-PR | CCCAGCCTCCGTTATCC |
| mmu-*Bax*-qRT-PCR-PF | AGGATGCGTCCACCAAG |
| mmu-*Bax*-qRT-PCR-PR | AAGTAGAAGAGGGCAACCAC |
| ssc-*cPLA2*-qRT-PCR-PF | CCCACAAGTTCACGG |
| ssc-*cPLA2*-qRT-PCR-PR | CCATACTAAATCGGAGGT |
| ssc*-iPLA2*-qRT-PCR-PF | CAGCCAGATTCACAGCAAAG |
| ssc-*iPLA2*-qRT-PCR-PR | AGGCAGAGCAGGTGGTCAT |
| ssc-*sPLA2*-qRT-PCR-PF | TGTTGGCTGTTCTGCTC |
| ssc-*sPLA2*-qRT-PCR-PR | GTGCTCCTTGTTGTATGG |
| ssc-*TRPC1*-qRT-PCR-PF | GATGTGCGGGAGGTGAA |
| ssc-*TRPC1*-qRT-PCR-PR | GGAATGTCGGAGGCTGTC |
| ssc-*TRPC6*-qRT-PCR-PF | AATACCGAAGAAGTAGAGGC |
| ssc-*TRPC6*-qRT-PCR-PR | CGAGGACCACAAGGAAC |
| ssc-*TRPM7*-qRT-PCR-PF | GGGGTTCTCATTCCTAC |
| ssc-*TRPM7*-qRT-PCR-PR | ACAGTGCCATCATCCA |
| ssc-*12-LOX*-qRT-PCR-PF | GATGGAGAG GTA CGTGAC |
| ssc-*12-LOX* -qRT-PCR-PR | AGTAGTTAGCCCCTTGG |
| ssc-*PTGS2*-qRT-PCR-PF | GAATCATTCACCAGGCAAATTG |
| ssc-*PTGS2*-qRT-PCR-PR | TCTGTACTGCGGGTGGAACA |
| ssc-*LHR*-qRT-PCR-PF | GAGTGACTGGGATTATGACTATGGT |
| ssc-*LHR*-qRT-PCR-PR | GCAATGAGTAGCAGGTAGAGCC |
| ssc-*CYP19A1*-qRT-PCR-PF | AAGAAGGGTCACAACAAG |
| ssc-*CYP19A1*-qRT-PCR-PR | AAGAAAGCCAGTGAGCAG |
| ssc-*AREG*-qRT-PCR-PF | GCACCTGGAAGCAGTAAC |
| ssc-*AREG*-qRT-PCR-PR | TTTTGATAAATCGCTGTGGA |
| ssc-*EREG*-qRT-PCR-PF | GTGGCTCAAGTGTCAATA |
| ssc-*EREG*-qRT-PCR-PR | CAAGAATCACGGTCAAA |
| mmu-*Ptgs2*-qRT-PCR-PF | CCTTCCTCCCGTAGCAG |
| mmu-*Ptgs2*-qRT-PCR-PR | CCCAGGTCCTCGCTTAT |
| mmu*-Lhr*-qRT-PCR-PF | CGCCCGACTATCTCTCACCTA |
| mmu-*Lhr*-qRT-PCR-PR | GACAGATTGAGGAGGTTGTCAAA |
| mmu-*Cyp19a1*-qRT-PCR-PF | ATGTTCTTGGAAATGCTGAACCC |
| mmu-*Cyp19a1*-qRT-PCR-PR | AGGACCTGGTATTGAAGACGAG |
| mmu-*Areg*-qRT-PCR-PF | GAGGTTTCCACCATAAGC |
| mmu-*Areg*-qRT-PCR-PR | CACCGTTCACCAAAGTAA |
| mmu-*Ereg*-qRT-PCR-PF | GCTGCTTTGTCTAGGTTC |
| mmu-*Ereg*-qRT-PCR-PR | TTCTTTGCTCAAGGGTT |
| mmu-*β-actin*-qRT-PCR-PF | GGCACCACACCTTCTACAATG |
| mmu-*β-actin*-qRT-PCR-PR | GGGGTGTTGAAGGTCTCAAAC |
| ssc-*β-actin*-qRT-PCR-PF | CCAGGTCATCACCATCGG |
| ssc*-β-actin*-qRT-PCR-PR | CCGTGTTGGCGTAGAGGT |
| mtDNA-PF | AACCTGGCACTGAGTCACCA |
| mtDNA-PR | GGGTCTGAGTCTATATATCATGAAGAGAAT |
| sgRNAs1*-Ggt1* | CGACCACGTGTACTCCAGGG |
| sgRNAs2-*Ggt1* | CCGCCGCCCTGGAGTACACG |
| ssc-si-*GGT1-01* | GGCCTTTGCATATCCACTC |
| ssc-si-*GGT1-02* | GAGGTGGTCCGCAACATGA |
| ssc-si-*SRSF1* | GCCCAGAAGTCCAAGTTAT |
| ssc-si-*hnRNPH1* | CAAGTCAAACAACGTTGAA |

Note: PF was upstream primer. PR was downstream primer. The part highlighted with grey was enzyme site induced.

**Table S2**. Information of antibodies used in Western blotting, Immunofluorescence, Co-IP, and RIP.

| **Antigen** | **Catalog number** | **WB** | **IHC** | **IF** | **Producer** |
| --- | --- | --- | --- | --- | --- |
| GGT1 | sc-166908 | 1:1000 | 1:200 | 1:200 | SANTA |
| TMCO1 | ab238768 | 1:1000 | 1:200 | 1:200 | Abcam |
| PCNA | A12427 | 1:1000 | Not applied | Not applied | ABclonal |
| BCL-2 | A0208 | 1:1000 | Not applied | Not applied | ABclonal |
| BAX | A19684 | 1:1000 | Not applied | Not applied | ABclonal |
| cPLA2 | A1678 | 1:1000 | Not applied | Not applied | ABclonal |
| AREG | A1860 | 1:1000 | Not applied | Not applied | ABclonal |
| EREG | A16372 | 1:1000 | Not applied | Not applied | ABclonal |
| CYP19A1 | A2161 | 1:1000 | Not applied | Not applied | ABclonal |
| PGR | A19697 | 1:1000 | Not applied | Not applied | ABclonal |
| LHR | A19020 | 1:1000 | Not applied | Not applied | ABclonal |
| TRPC1 | ab192031 | 1:1000 | 1:200 | Not applied | ABclonal |
| β-actin | AC026 | 1:500000 | 1:200 | Not applied | ABclonal |
| DDDDK-Tag | AE063 | 1:1000 | 1:200 | 1:100 | ABclonal |
| HA-Tag | AE008 | 1:1000 | 1:200 | 1:100 | ABclonal |
| PTGS2 | A1253 | 1:1000 | 1:200 | 1:100 | ABclonal |
